# Early Compound Nutritional Intervention Improves Hair Follicle Development in Northwest Xizang White Cashmere Goats: An Analysis Based on Skin Multi-Omics

**DOI:** 10.3390/ani16172756

**Published:** 2026-09-02

**Authors:** Yiming Liu, Xiaolong Wu, Jiaoyang A, Zepeng Duan, Meng Yao, Siying Meng, Hui Zhao, Jie Liu, Yunxia Guo, Zili Ren, Yujiang Wu, Yueqin Liu, Hongna Wang

**Affiliations:** 1 College of Life Science and Food Engineering, Hebei University of Engineering, Handan 056000, China; l760407841@163.com (Y.L.); wxl18835202424@163.com (X.W.);; 2 Shijiazhuang Academy of Agriculture and Forestry Sciences, Shijiazhuang 050000, China; 3 College of Animal Science and Technology, Hebei Agricultural University, Baoding 071000, China; 4 College of Animal Science, Xizang Agricultural and Animal Husbandry University, Nyingchi 860006, China; 5 Institute of Animal Husbandry and Veterinary, Xizang Autonomous Regional Academy of Agricultural Sciences, Lhasa 850000, China

**Keywords:** Northwest Xizang White Cashmere goat, secondary hair follicles, early compound nutritional intervention, multi-omics

## Abstract

The Northwest Xizang White Cashmere goat has a high global reputation for good goat cashmere germplasm characteristics, and its cashmere industry is the economic pillar of the herdsmen in the northwest plateau of Xizang. Cashmere is produced by the secondary hair follicles of the skin, and the key period of hair follicle development is from birth to 3 months old. This study aimed to explore how early compound nutritional intervention affects lamb growth performance and secondary hair follicle development. We assessed skin follicle density and performed skin transcriptomic and multi-omics analyses to clarify this regulatory effect. The trial was performed at the Northwest Xizang White Cashmere Goat Breeding Farm, Ritu County, Ngari region, Xizang. Thirty newborn single lambs of comparable birth status were randomly divided into control and early compound nutritional intervention groups (*n* = 15 per group). The results showed that early compound nutritional intervention significantly increased the secondary hair follicle density, total hair follicle density, and secondary/primary hair follicle (S/P) value of lambs (*p* < 0.05). However, there was no significant difference in the average cashmere fineness between the two groups (*p* > 0.05). The transcriptome, proteome, and metabolomics analyses of the skin revealed candidate genes related to hair follicle development and the regulation of cashmere fineness: keratin structural genes (KRT10, KRT18, KRT26), cell proliferation and adhesion-related regulatory genes (ITGB2, CSF1R, AKT1), and extracellular matrix coding genes (COL6A1, COL6A2, COL6A6). Moreover, the results revealed that key proteins related to hair follicle development include RBP4, KAP13-3, KRTAP3-1, KRTAP27-1, DKK3, ITGB2, and DUSP23. The differentially expressed metabolites, 15-Deoxy-delta12,14-Prostaglandin J2 and Thromboxane B2 (TXB2), modulate prostaglandin-associated pathways; altered pathway activity subsequently regulates the expression of hair follicle functional genes and key proteins, which may in turn indirectly regulate hair follicle growth and development, thereby affecting hair follicle density and, by extension, cashmere production performance. Therefore, early compound nutritional intervention of Northwest Xizang White Cashmere goats promotes the growth and development of secondary hair follicles. The identified candidate genes and key proteins involved in secondary hair follicle development provide a reference for exploring the mechanism of hair follicle development.

## Simple Summary

The high-quality cashmere produced by Northwest Xizang White Cashmere goats serves as the primary source of income for local herdsmen. The cashmere-producing hair follicles experience their critical developmental window within the first three months after lamb birth. This study investigated the effects of early compound nutritional intervention on lamb growth and hair follicle development. The results revealed that early compound nutritional intervention markedly elevated the secondary hair follicle density, total hair follicle density, and the ratio of secondary to primary hair follicles in lambs, whereas no significant difference in average cashmere fineness was detected between the compound nutritional intervention group and the control group. Transcriptomic, proteomic and metabolomic analyses of skin tissues identified a panel of candidate genes associated with hair follicle development and cashmere fineness regulation, including KRT10, KRT18, KRT26, ITGB2, CSF1R, AKT1, COL6A6, COL6A2 and COL6A1. Multiple core proteins closely involved in hair follicle development were also screened out, namely RBP4, KAP13-3, KRTAP3-1, KRTAP27-1, DKK3, ITGB2 and DUSP23. Two differentially expressed metabolites, 15-Deoxy-delta12,14-Prostaglandin J2 and TXB2, may indirectly mediate hair follicle growth and development by modulating prostaglandin derivatives through relevant signaling pathways, which further alters hair follicle density and ultimately influences cashmere yield.

## 1. Introduction

The Northwest Xizang White Cashmere goat is a famous cashmere-meat dual-purpose global goat variety with strong adaptability and resistance to roughage [1]. This ancient goat is a valuable resource grazed in the harsh alpine ecological environment of China all year round. The cashmere produced is fine and white, a high-end raw material for the international cashmere textile industry. Globally, the Northwest Xizang White Cashmere goat has a high reputation for high-quality cashmere germplasm, and its cashmere industry is the main economic pillar for herdsmen on the Northwest Xizang Plateau.

Goats have been raised in Xizang for thousands of years, and the Northwest Xizang White Cashmere goats account for ~41% of the total number of cashmere goats [2]. The white cashmere goats of Ritu and Nima counties in Xizang are known as the origin of cashmere goats in Kashmir. Such high-quality cashmere can sell at higher prices, thereby creating significant benefits for residents. However, backward feeding methods, severe grassland degradation [3], and a long withering period limit the nutritional intake of these goats, resulting in low production and cashmere yield.

Hair follicles are an important skin accessory organ, and their basic structure is similar across different animals. The unique structure and ability of the hair follicle to undergo periodic growth make it vital to hair growth. The hair follicle can be divided into four parts from top to bottom, which are the funnel, isthmus, wheel, and the hair bulb [4]. However, the hair follicles of cashmere goat skin are categorized into primary and secondary hair follicles. The primary hair follicles, which occur earlier, generate wool, and the secondary follicles generate cashmere. The primary hair follicle has a rich accessory structure, and the secondary hair follicle has no sweat glands or erector muscle. Additionally, secondary hair follicles exhibit periodic growth and self-renewal.

The hair follicle activity cycle has three stages: growth, regression, and telogen, in a closely related and coordinated process regulated by various genes and actors [5]. This continuous bag-like epithelium formed by epidermal cells grows through three stages: anagen, catagen, and telogen, in which its morphology and metabolic activity change dramatically. The activity cycle of the primary hair follicle is long, and the periodic change is small, while secondary hair follicles experience three periods: growth, degeneration, and dormancy in one year. Cashmere is only produced by secondary hair follicles. Due to the cyclical nature of hair follicles, cashmere goats experience seasonal hair-shedding during the telogen and growth phases. Hair follicles often regenerate before the end of the previous cashmere generation cycle and begin to prepare for the next growth cycle [6]. Thus, the secondary hair follicles of cashmere goats are in a state similar to “resting” from December to March, during which the hair shafts gradually become inactive [7]. In April, their epidermis gradually thickens, and the hair follicles begin to elongate and rebuild. By August and September, the density of primary and secondary hair follicles reaches the maximum. In other studies, the epidermal germinal layer of Liaoning cashmere goats began to move and produce cashmere from March to May, grew vigorously from July to November, and peaked in September [7,8,9].

Chinese cashmere goat breeds are relatively abundant, most of which are distributed in desert and semi-desertified areas. Traditional grazing cashmere goat breeding is one of the important reasons for grassland degradation and desertification. Thus, barn and semi-house feeding systems, which balance ecological conservation and livestock production, have been widely adopted to mitigate the conflict between ecological-environment protection and the development of cashmere goat husbandry. Barn feeding generates more cashmere yield in the Liaoning cashmere goats than other grazing goats [10]. Moreover, the cashmere content and natural length are also better. It can be seen that the feeding method of cashmere goats can promote the potential of cashmere production.

Dietary nutrition is a key factor affecting the production of cashmere goats. Therefore, increasing the dietary protein of Liaoning cashmere goats during the cashmere growth period can significantly increase the length and yield of cashmere short fibers. At the same time, restricting the high-protein level within a certain range will preserve the cashmere length [11]. For instance, adding rumen cysteine and methionine to the diet significantly enhanced the activity of secondary hair follicles and improved cashmere length and yield [12]. In addition, precisely supplementing and feeding pregnant ewes according to the nutritional requirements of cashmere goats increased the cashmere yield and length [7]. Nevertheless, most existing studies have focused on cashmere-growing adult goats rather than newborn lambs during the critical window of postnatal secondary hair follicle development. It is unclear whether early nutritional intervention regulates secondary hair follicle morphogenesis in neonatal Northwest Xizang White Cashmere goat lambs. We thus hypothesized that early compound nutritional intervention could optimize lamb nutrient status, promote secondary hair follicle development, and improve cashmere-producing potential.

The Northwest Xizang White Cashmere goats are mainly raised by grazing. However, the grassland is degraded, and in winter and spring, withering periods are long, making the nutritional intake of white cashmere goats in Northwest Xizang inadequate for their growth needs, hence, low cashmere yield. Spring is the birth period of lambs, and the nutrition of the lambs at this early stage plays a decisive role in their later growth and development. Specifically, the nutritional status of lambs within 3 months of birth significantly affects hair follicle development. Thus, this study aimed to investigate the effects of an early compound nutritional intervention (15–75 days of age) on lamb growth performance, serum biochemistry, and hair follicle development, and to systematically screen differentially expressed genes, differentially expressed proteins, and differential metabolites in skin tissues via integrated transcriptomic, proteomic, and metabolomic analyses, together with serum metabolome and fecal microbiome profiling, thereby providing a theoretical basis for targeted nutritional strategies to optimize cashmere quality and yield in alpine grazing areas of Northwest Xizang.

## 2. Materials and Methods

### 2.1. Experimental Design

This experiment was performed at the Northwest Xizang White Cashmere Goat Breeding Farm in Ritu County, Ngari region, Xizang, from April to June 2025. The farm is located at an average altitude of about 4800 m. All lambs were raised in semi-open pens under natural ambient temperature and light. Immediately after birth, lambs were ear-tagged, and their birth dates, birth types (single or twin), sex, and birth weights were recorded. At approximately 14 days of age, 30 single lambs with uniform birth dates and initial body weights (4.46 ± 0.80 kg) were selected and randomly divided into 2 groups (*n* = 15 per group; 8 males and 7 females): a control (D) group and a compound nutritional intervention (S) group. Throughout the lactation period, all lambs remained with their dams for suckling. In addition to maternal milk, the S group was provided with a specialized lamb starter feed ad libitum, whereas the D group received no supplementation. The study consisted of a 7-day acclimation period followed by a 60-day formal trial.

### 2.2. Feeding Management

Starting from 20 days of age, all lambs were allowed to graze with their dams. The control group relied exclusively on natural grazing and maternal milk throughout the experimental period. In contrast, lambs in the compound nutritional intervention group were moved to separate enclosures for supplementation following each grazing session. These lambs were provided ad libitum access to concentrate pellets twice daily (at 10:00 and 18:30) The supplementary diet was fortified with rumen-protected methionine (0.5 g/day per head) and crushed Lactobacillin tablets. The rumen-protected methionine used in this trial was a commercial product (Ougubao, mixed feed additive DL-methionine), purchased from Ougu Biotechnology Hebei Co., Ltd., Shijiazhuang, China. The guaranteed methionine content was ≥58%. Lactobacillin tablets were purchased from Jiangzhong Pharmaceutical Co., Ltd., Nanchang, China. The main active ingredient was lactobacillin (0.2 g per tablet).

To ensure true ad libitum intake, the amount of feed offered was adjusted daily based on individual consumption to maintain consistent orts. During the initial induction phase, a specific proportion of milk powder was top-dressed on the pellets to encourage solid feed intake (Table 1). All lambs had free access to fresh water throughout the study.

### 2.3. Experimental Diet

The pellet feed used in this experiment was 901 lamb compound feed produced by Wuwei Haimu Biotechnology Co., Ltd. (Wuwei, Gansu, China). Table 2 shows the composition and nutritional components of the basic diet raw materials.

### 2.4. Indicators and Methods of Determination

At the beginning and the end of the experiment, the shavers were used to shave a 5 × 5 cm^2^ square at the same part of the lamb (the posterior edge of the left scapula was selected uniformly) for wool and cashmere collection.

Skin sample collection: A piece of skin (about 1 cm^2^) was cut with surgical scissors, then washed with normal saline and cleaned with RNase water. After the water was dried, the sample was placed in a 2 mL cryopreservation tube containing RNA preservation solution and snap-frozen in liquid nitrogen, then stored at −80 °C (for subsequent skin transcription and metabolomics analysis). An additional skin sample was collected using a 10 mm diameter biopsy punch and bisected with ophthalmic scissors: one half was snap-frozen for proteomic analysis, while the other half was fixed in 4% paraformaldehyde for histological evaluation of hair follicles.

Wool length: The wool samples collected from the lamb were stretched naturally on the black flannel board, and their lengths were measured and recorded with a ruler.

Fineness of cashmere: The Australian OFDA 2223 optical fiber diameter analyzer was used to measure cashmere fineness, with more than 2000 fibers counted per measurement, and the average of three measurements for each sample was used as the final fineness.

Follicle population: The skin samples collected and stored in a glass bottle filled with tissue fixative were sent to Nanjing Jichu Biomedical Technology Co., Ltd. (Nanjing, China) for the detection of the skin hair follicle density. The specific experimental steps were as follows: first, formalin was used for tissue dehydration for 1 h, followed by gradient dehydration with ethanol, xylene transparent treatment, and paraffin embedding. The samples were cut into 7 μm sections, HE-stained, and finally observed under a microscope after sealing with neutral resin and photographed through transverse sections of skin hair follicles.

### 2.5. Multi-Omics Experimental Procedures and Bioinformatics Analysis

Skin transcriptome, metabolome, and proteome: The skin tissue samples in the frozen tube were sent to Nuohe Zhiyuan Biotechnology Co., Ltd. (Beijing, China) for skin transcriptome, metabolomics, and proteomics analysis. Fifteen lambs were included in each group for the feeding trial. Five lambs were randomly selected from each group for phenotypic measurement and proteomic and metabolomic analyses.

Total RNA was extracted and subjected to quality assessment, library construction, and Illumina paired-end sequencing. After quality control of the raw reads, clean reads were aligned to the reference genome using HISAT2, and gene expression levels were quantified. Differentially expressed genes (DEGs) were identified using DESeq2 (v1.20.0). The screening threshold was set as |log2(FoldChange)| ≥ 1 and adjusted *p*-value (padj) ≤ 0.05, followed by GO and KEGG enrichment analyses and GSEA to investigate their potential biological functions.

Protein samples were extracted, quantified, enzymatically digested, and analyzed by LC-MS/MS using a data-independent acquisition (DIA) strategy. Protein identification and quantification were performed using DIA-NN. Differentially expressed proteins (DEPs) were identified based on the criterion of |fold change| > 1.5 (up-regulated by >1.5-fold or down-regulated by <0.67-fold) and *p*-value < 0.05, followed by GO and KEGG functional enrichment and protein–protein interaction analysis.

Untargeted metabolomic profiling was performed by mass spectrometry. Raw data were converted to mzXML format using ProteoWizard (v3.0.22107) and processed using XCMS for peak extraction, alignment, and retention-time correction. Metabolites were identified using relevant databases. PCA and PLS-DA were performed for multivariate analysis. Differential metabolites were screened based on the criteria of variable importance in projection (VIP) > 1.0, fold change (FC) > 1.2, and *p*-value < 0.05, followed by hierarchical clustering, correlation analysis, and KEGG pathway enrichment.

Fecal microbiome composition was characterized using 16S rRNA gene sequencing. Following quality control and taxonomic assignment, alpha- and beta-diversity analyses were performed to evaluate microbial richness, diversity, and community structure. PCA, PCoA, NMDS, and ANOSIM were used to assess differences in microbial community composition between groups, followed by analysis of differential microbial taxa.

### 2.6. Data Processing and Analysis

The experimental data were preliminarily sorted in Excel, and the statistical analysis was performed using SPSS 27.0 for an independent-samples *t*-test (Lambs were artificially matched before grouping; birth weight and sex ratio were well-balanced between the two groups with no significant baseline difference). The data were expressed as mean ± standard error (SEM), and differences were considered statistically significant when *p* < 0.05.

For the transcriptomic dataset, raw reads were quality-controlled using FastQC (v0.11.9), and clean reads were aligned to the reference genome using HISAT2. Gene expression levels were quantified using feature counts, and differentially expressed genes (DEGs) were identified using DESeq2. GO and KEGG enrichment analyses were performed using clusterProfiler (v3.8.1), and gene set enrichment analysis (GSEA) was conducted using the GSEA software (v3.0).

For the proteomic dataset, raw DIA data were processed using DIA-NN for protein identification and quantification. Differentially expressed proteins (DEPs) were identified by *t*-test with the threshold of |fold change| > 1.5 and *p* < 0.05. GO and KEGG functional enrichment analyses were performed using clusterProfiler, and protein–protein interaction (PPI) networks were constructed using the STRING database and visualized in Cytoscape (v3.9.1).

For the metabolomic dataset, raw mass spectrometry data were converted to mzXML format using ProteoWizard (v3.0.22107), and peak extraction, alignment, and retention-time correction were performed using XCMS. Metabolite identification was conducted by matching against public databases (HMDB, METLIN, and KEGG). Multivariate statistical analyses, including principal component analysis (PCA) and partial least squares discriminant analysis (PLS-DA), were performed using metaX (v1.4.19). Univariate analysis (*t*-test and fold-change calculation) was combined with VIP values to screen differential metabolites. Hierarchical clustering and correlation analyses were performed using the pheatmap and corrplot packages in R, and KEGG pathway enrichment was conducted using MetaboAnalyst (v5.0).

For the fecal microbiome dataset, 16S rRNA gene sequencing data were processed using QIIME2 (v2021.4). Alpha diversity (Shannon, Simpson, Chao1, and ACE indices) and beta diversity (Bray–Curtis distance) were calculated, and PCA, PCoA, NMDS, and ANOSIM were performed to assess differences in microbial community composition between groups. Differential microbial taxa were identified using LEfSe (v1.0.7) (LDA score > 3.0).

## 3. Results

### 3.1. Effect of Early Compound Nutritional Intervention on the Hair Follicle Density of Lambs

Two months of compound nutritional intervention did not significantly affect the primary hair follicle density of lambs in the experimental group (compared to the control) (*p* > 0.05). In contrast, it significantly increased the density of the secondary hair follicle, total hair follicle, and secondary hair follicle density/primary hair follicle density (S/P) ratio (*p* < 0.05). These three density indexes were nearly doubled after two months of compound nutritional intervention (Table 3).

**Figure 1:**
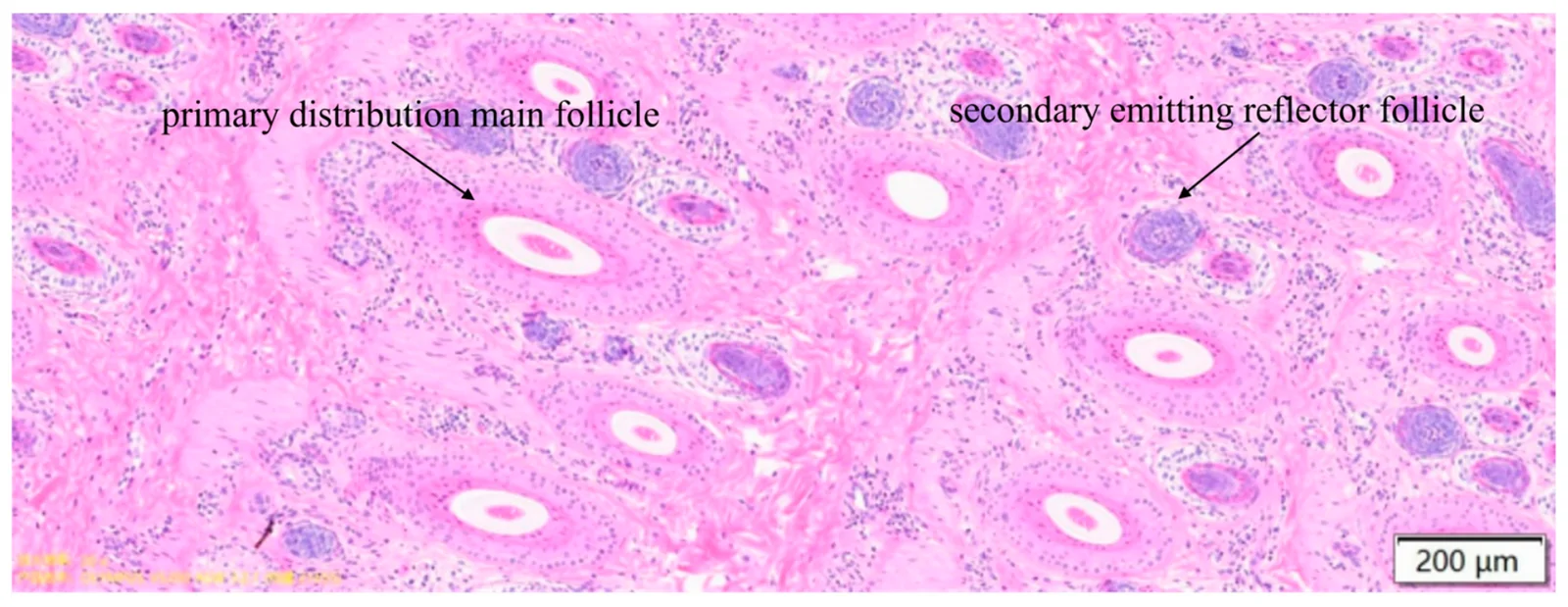
Cross-section scan of skin hair follicles of lambs in the Control group (60 times).

**Figure 2:**
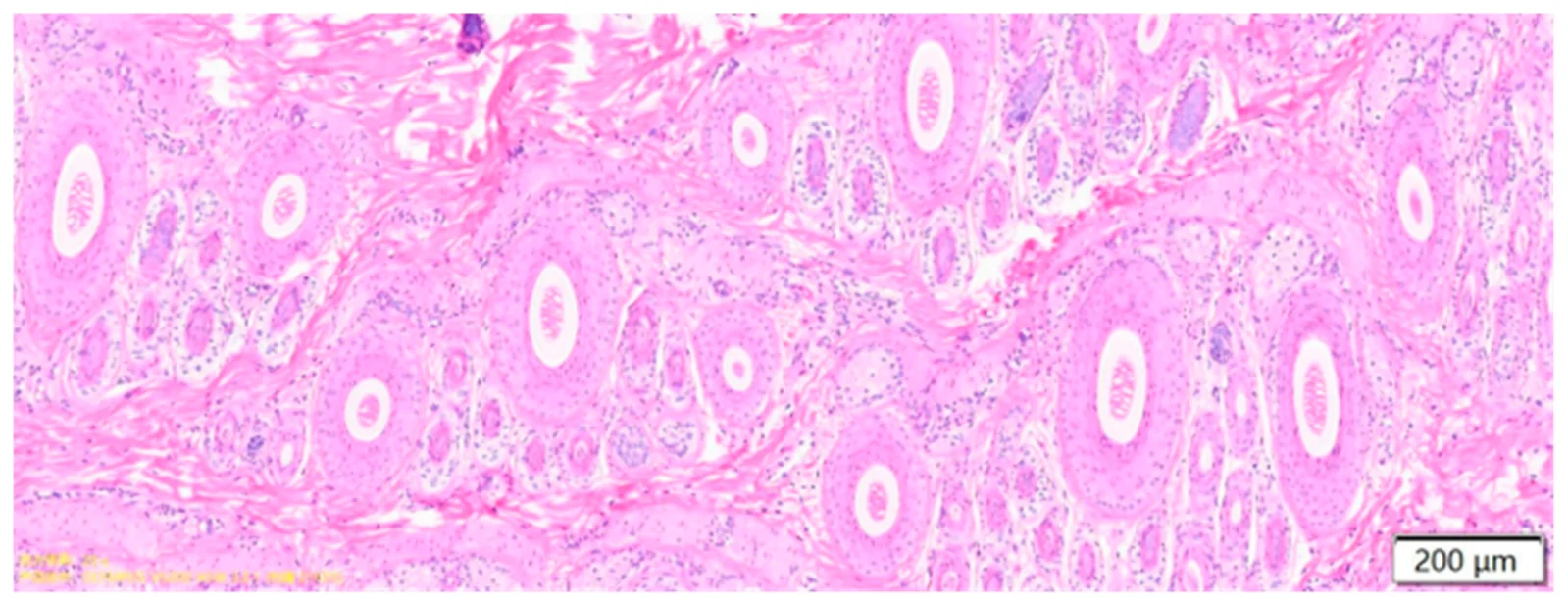
Cross-section scan of skin hair follicles of lambs in the experimental group (60 times).

The average S/P value of the two groups of lambs showed that the intercepted density was close to the corresponding hair follicle density scan map (60 times). As shown in Figure 1 and Figure 2, the primary hair follicles were larger, fewer in number, and had more cavities. In contrast, the secondary hair follicles were smaller and more numerous, with fewer cavities of different forms.

### 3.2. Effect of Early Compound Nutritional Intervention on Cashmere Performance of Lambs

At the end of the experiment, the lambs were approximately 10 weeks old, and the collected cashmere samples were insufficient to measure fineness. However, the cashmere length, cashmere yield, and other indicators could not be calculated. The two months of compound nutritional intervention raised the wool length to an average of 10.15 cm, significantly higher than the control group (*p* < 0.01) (Table 4). There was no significant difference in cashmere fineness between the two groups (*p* > 0.05).

### 3.3. Effects of Early Compound Nutritional Intervention on the Skin Transcriptome of Lambs

**Figure 3:**
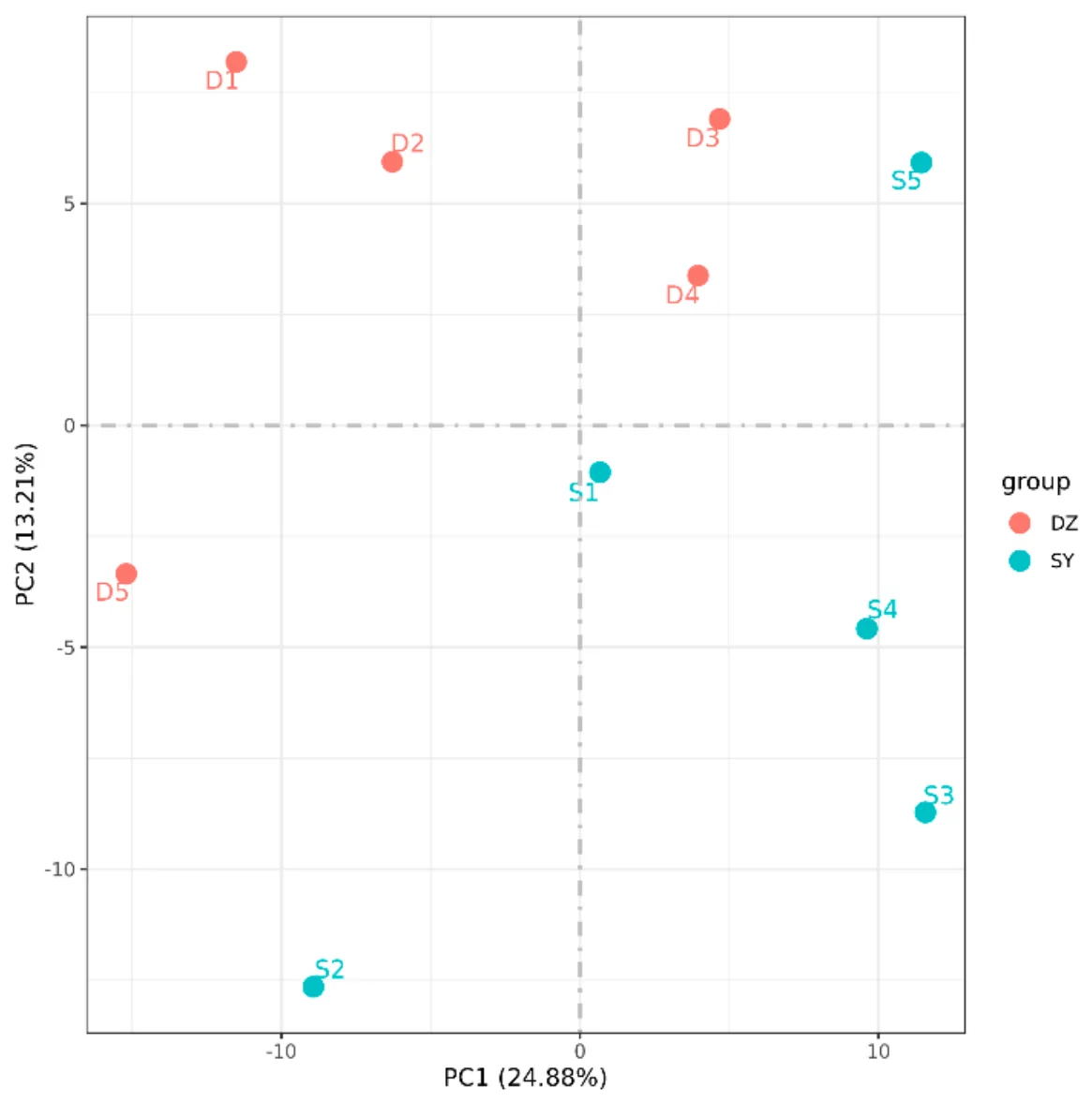
Principal component analysis plot.

**Figure 4:**
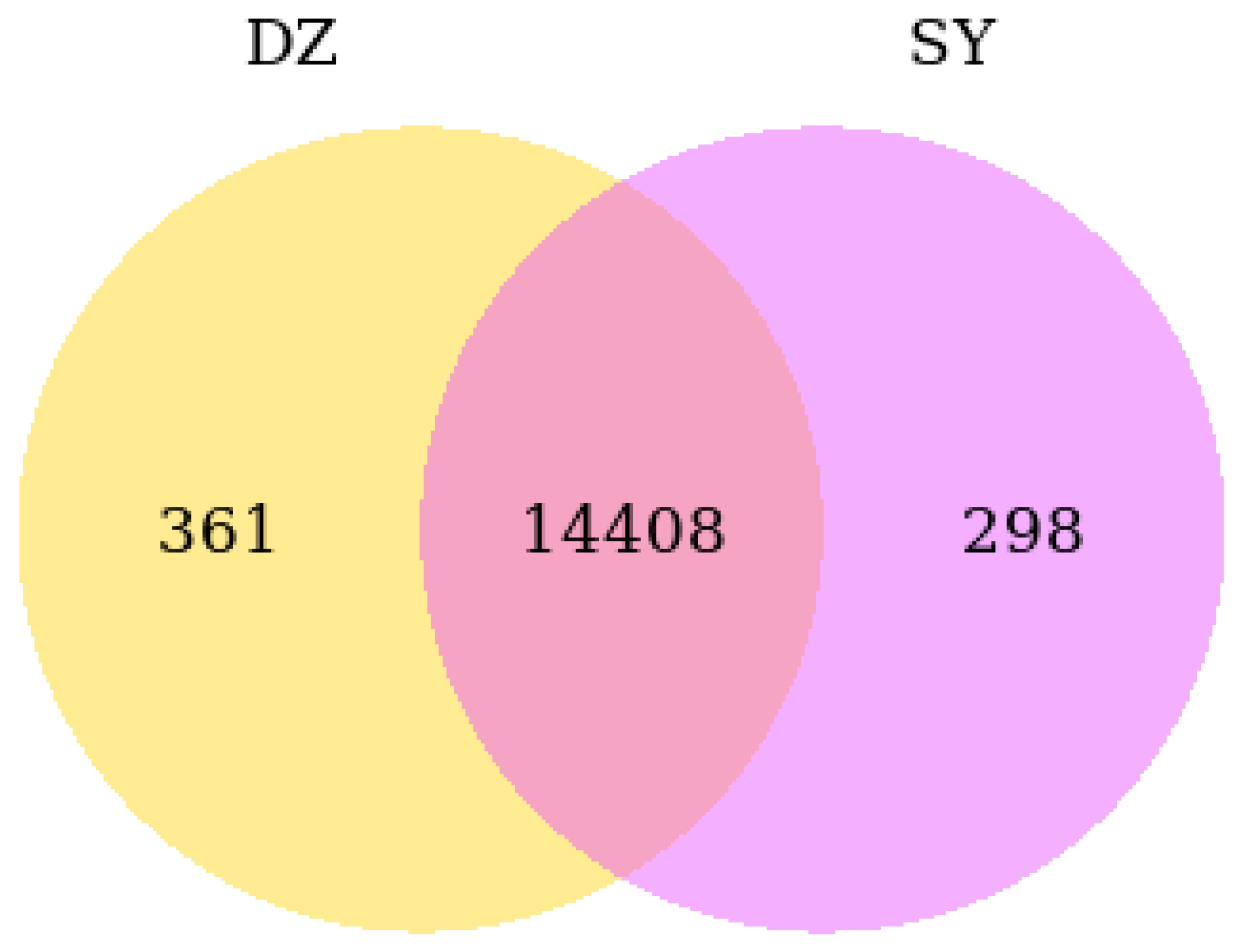
Co-expression Venn diagram. DZ represents the control group; SY represents the early compound nutritional intervention group.

The original data is filtered to retain only high-quality data for subsequent analysis. The Q30 (Q30 refers to the percentage of bases with a Qphred score > 30 in the total bases) of the lambs in the control and experimental groups after filtering was ~96% (the normal level is >85%), with a GC content of 49% (Table 5). The HISAT2 comparison of clean reads with the reference genome resulted in >92% similarity, indicating data reliability for subsequent analysis. Principal component analysis showed 24.88% and 13.21% contributions from the first and second principal components, respectively (Figure 3). The samples between the two groups are relatively dispersed, and those within the two groups are relatively aggregated. Removing individual samples improved the repeatability within each group. The number of co-expressed and uniquely expressed genes between the two groups was counted, and a co-expression Venn diagram was constructed. As shown in Figure 4, a total of 14,408 genes were co-expressed across the two groups; 361 genes were uniquely expressed in the control group, and 298 genes were uniquely expressed in the experimental group.

**Figure 5:**
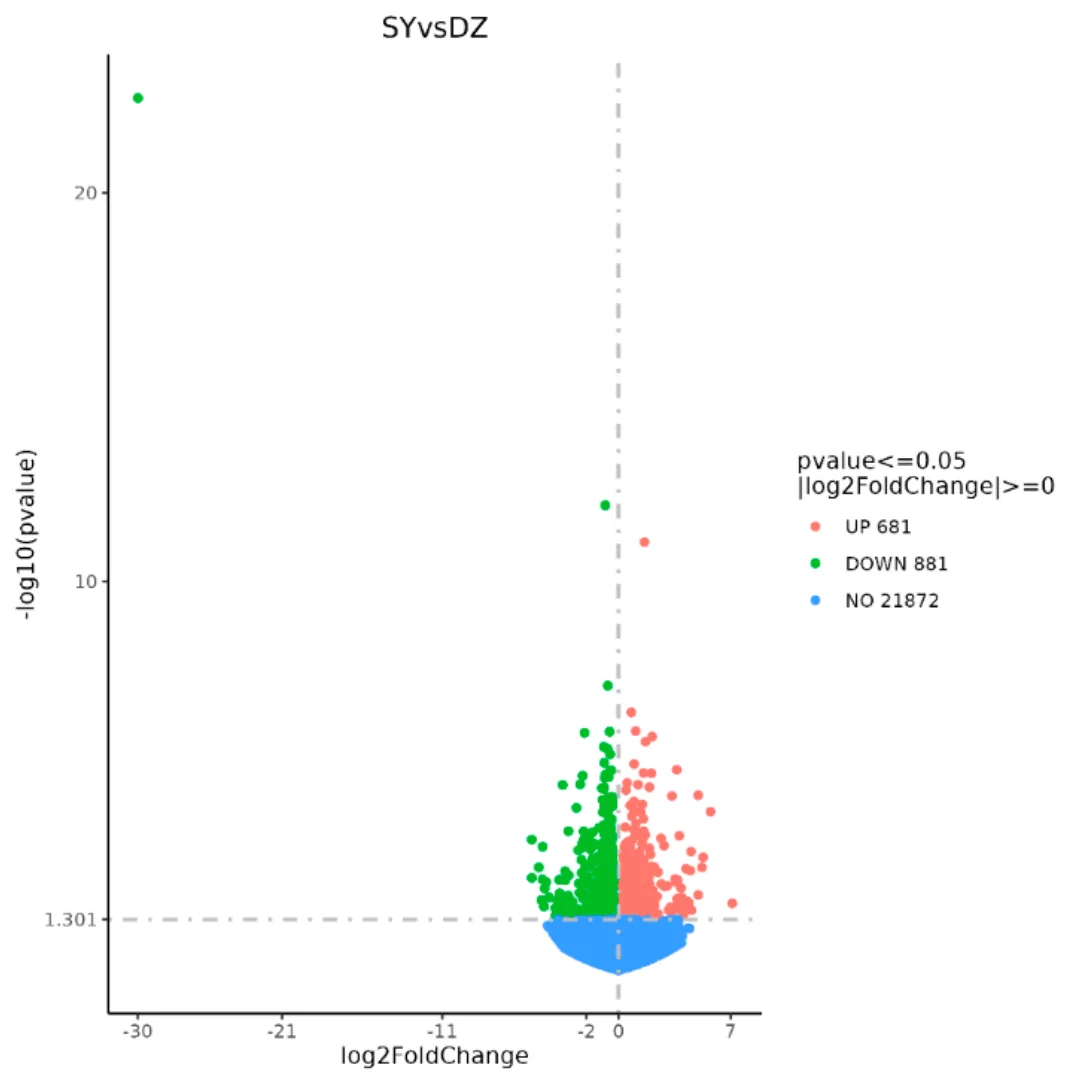
Differential gene volcano map. Red and green dots indicate up-regulated and down-regulated genes, respectively.

A total of 1562 significantly differentially expressed genes were identified, including 681 up-regulated and 881 down-regulated genes. The volcanic map shows the distribution of differential genes in the comparison combination (Figure 5).

**Figure 6:**
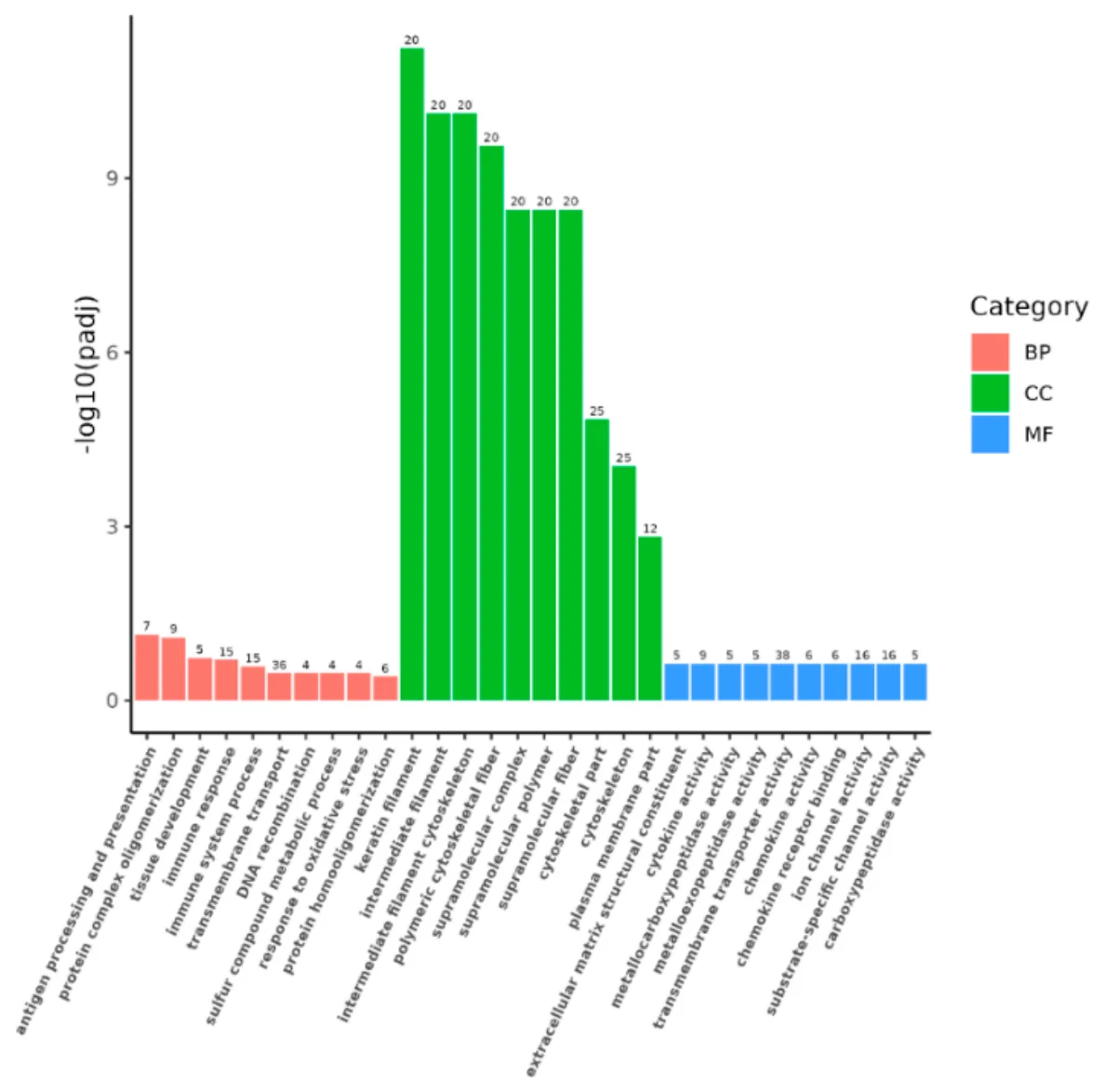
GO enrichment analysis histogram. The higher the value, the more significant the GO term. The values in the columns represent the number of differential genes enriched for the GO term; different colors (red, green, and blue) represent the three GO subclasses, namely biological process (BP), cellular component (CC), and molecular function (MF), respectively.

Enrichment analysis predicted key biological pathways, revealing the basic molecular mechanisms underlying these processes. The top 30 GO (Gene Ontology) terms showed that the cell components contained most of the significantly different genes (Figure 6). Further, the top ten differentially expressed genes in cell components included keratin filament, intermediate filament, intermediate filament cytoskeleton, polymeric cytoskeletal fiber, supramolecular complex, supramolecular polymer, supramolecular fiber, cytoskeletal part, cytoskeleton, and plasma membrane part.

**Figure 7:**
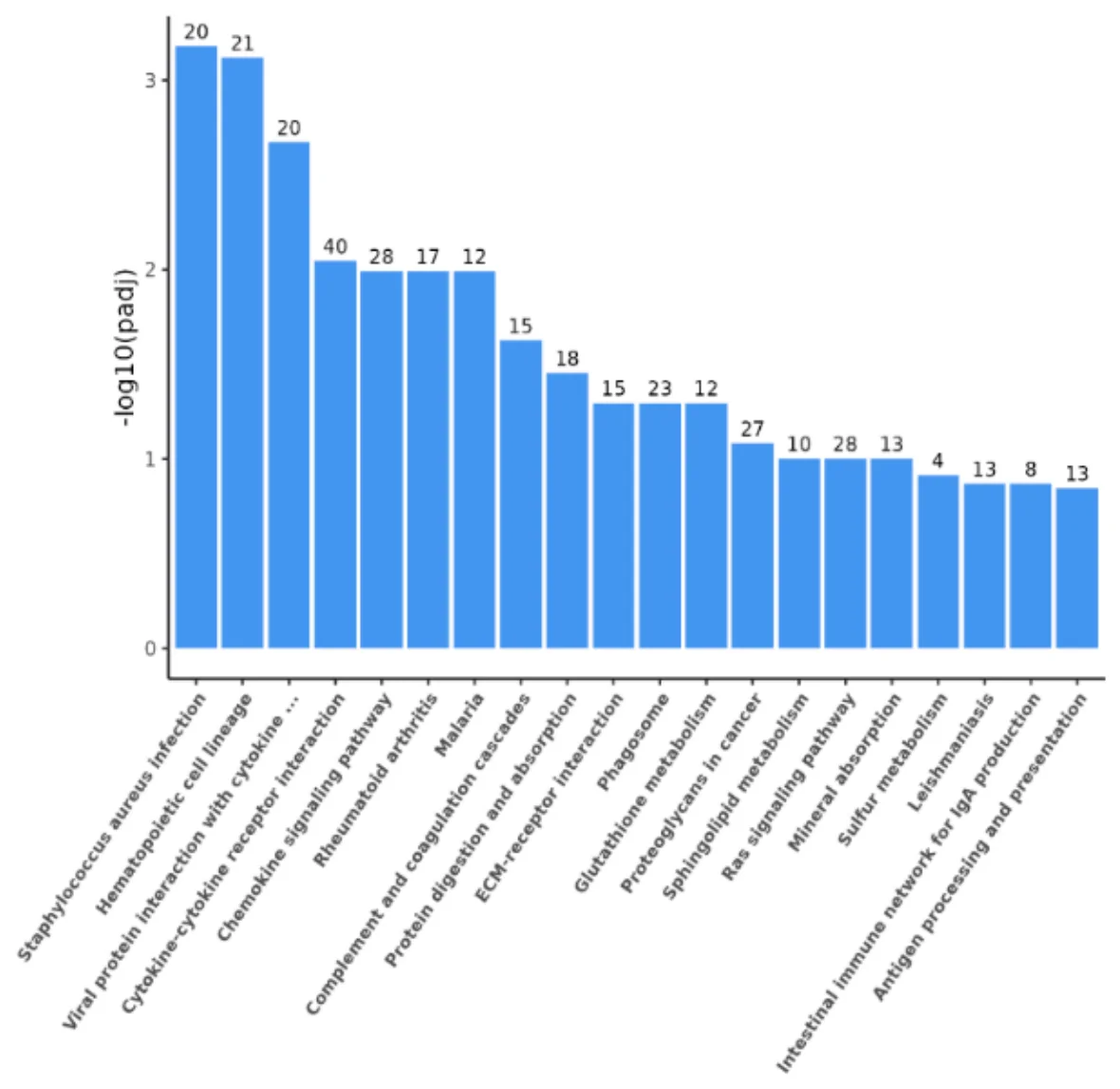
Histogram of enriched KEGG pathways. The values in the columns represent the number of differential genes that enriched the KEGG pathways.

For KEGG pathway enrichment, padj < 0.05 was the threshold for significant enrichment (Figure 7 and Table 6). The enriched pathways included Staphylococcus aureus infection, hematopoietic cell lineage, viral protein interaction with cytokine and cytokine receptor, cytokine–cytokine receptor interaction, chemokine signaling pathway, rheumatoid arthritis, malaria, complement and coagulation cascades, and protein digestion and absorption.

**Figure 8:**
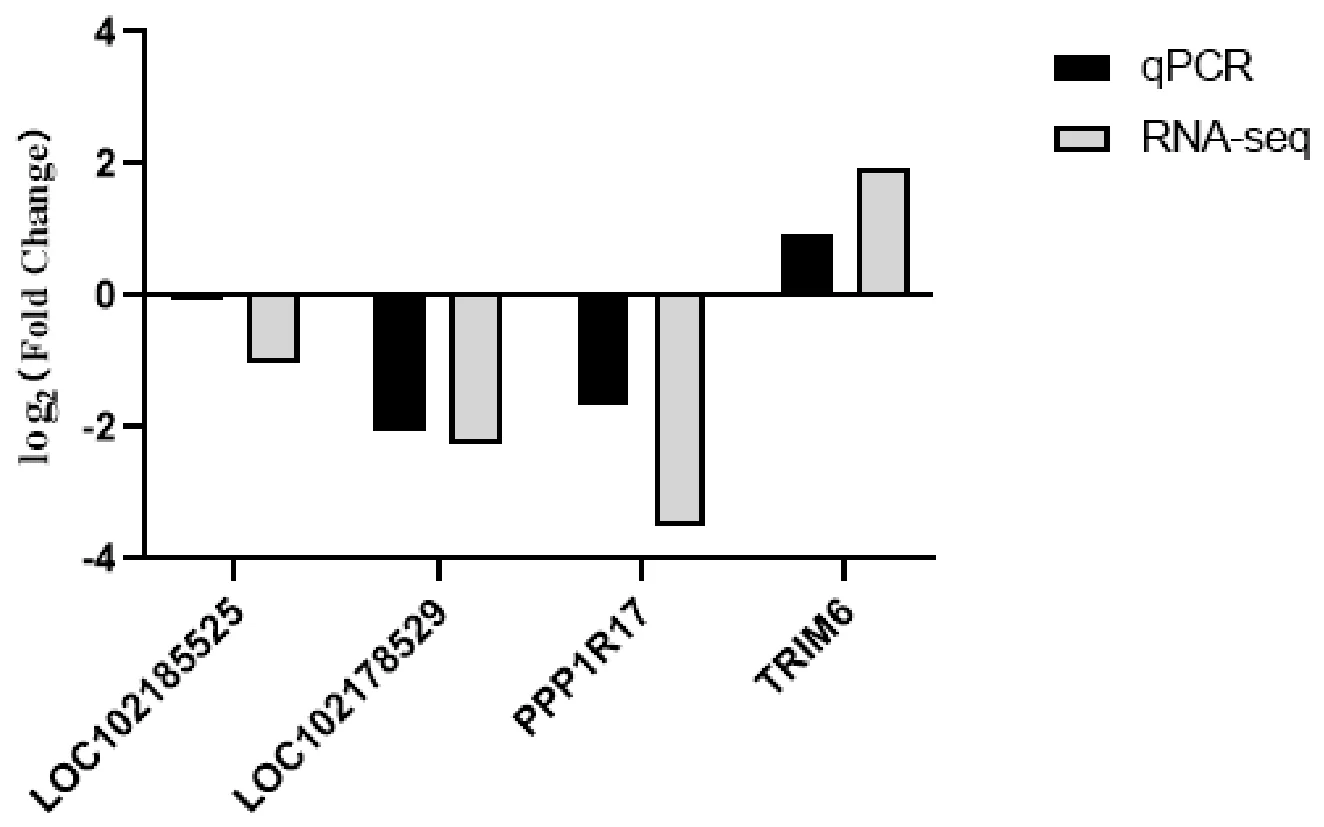
Comparison of mRNA qPCR results with RNA-seq results.

Thus, four genes related to the growth and development of cashmere goats [13] were randomly selected from the transcriptome differential gene expression results, with GAPDH as the internal reference. The primer sequences of these target genes and the reference gene are presented in Table A1 (Appendix A). Figure 8 shows that the differences among the four selected genes between the two groups were consistent with the transcriptomic results, indicating that the data analysis was reliable.

### 3.4. Effects of Early Compound Nutritional Intervention on the Skin Proteome of Lambs

**Figure 9:**
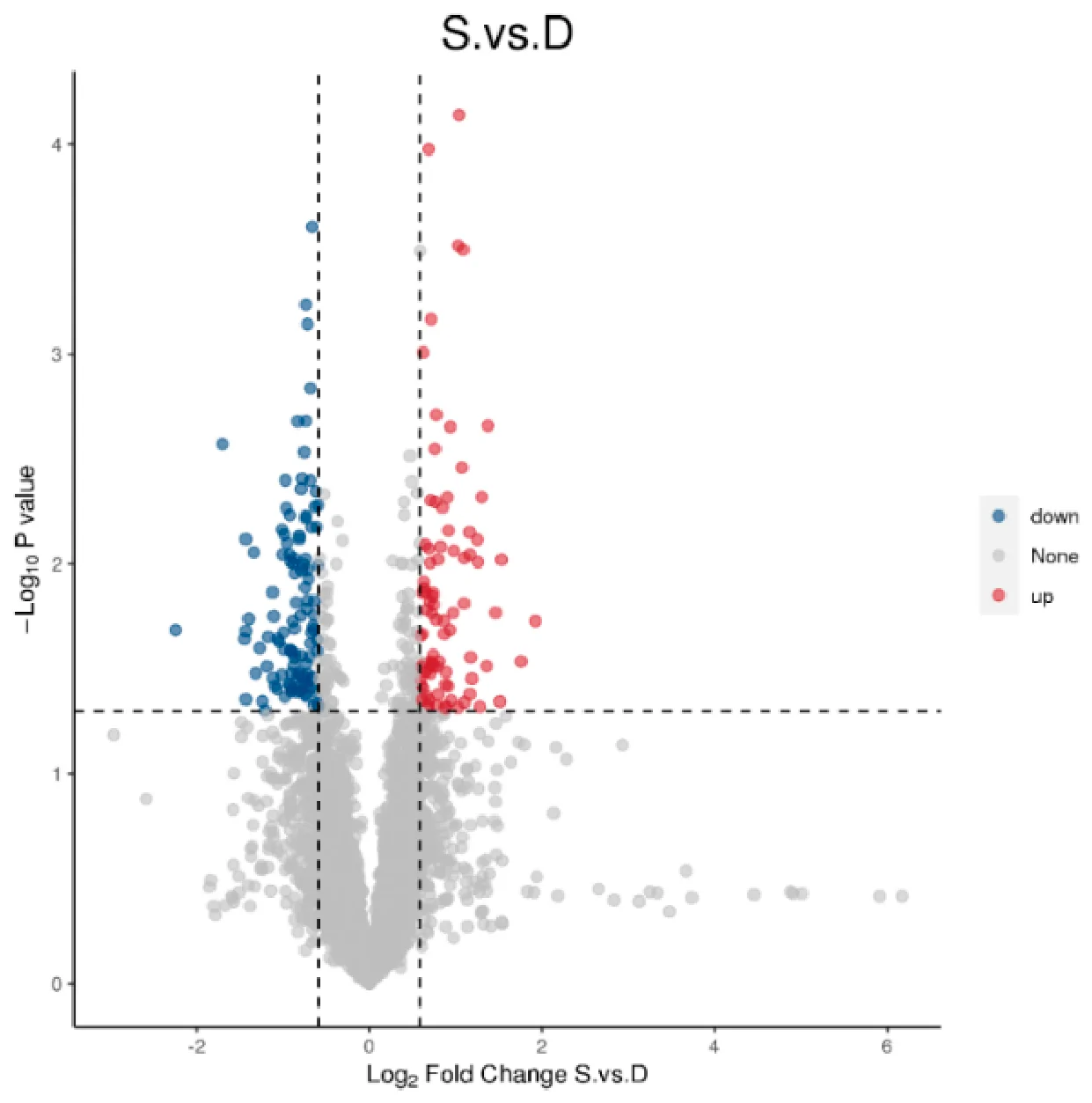
Volcano plot of differentially expressed proteins.

Data-independent acquisition (DIA) quantitative proteomics identified 9589 proteins across 10 samples from the two lamb groups. Differentially expressed proteins were identified following the criterion of |>1.5 times fold change (up-regulated by >1.5 times or down-regulated by <0.67 times) and *p*-value < 0.05|. The results showed that 198 proteins were significantly expressed (87 were significantly up-regulated and 111 were significantly down-regulated). The logarithm of the difference multiple of each protein was taken at the base of 2, and the negative value of the logarithm of the *p*-value was taken at the base of 10 to make a volcano map (Figure 9).

**Figure 10:**
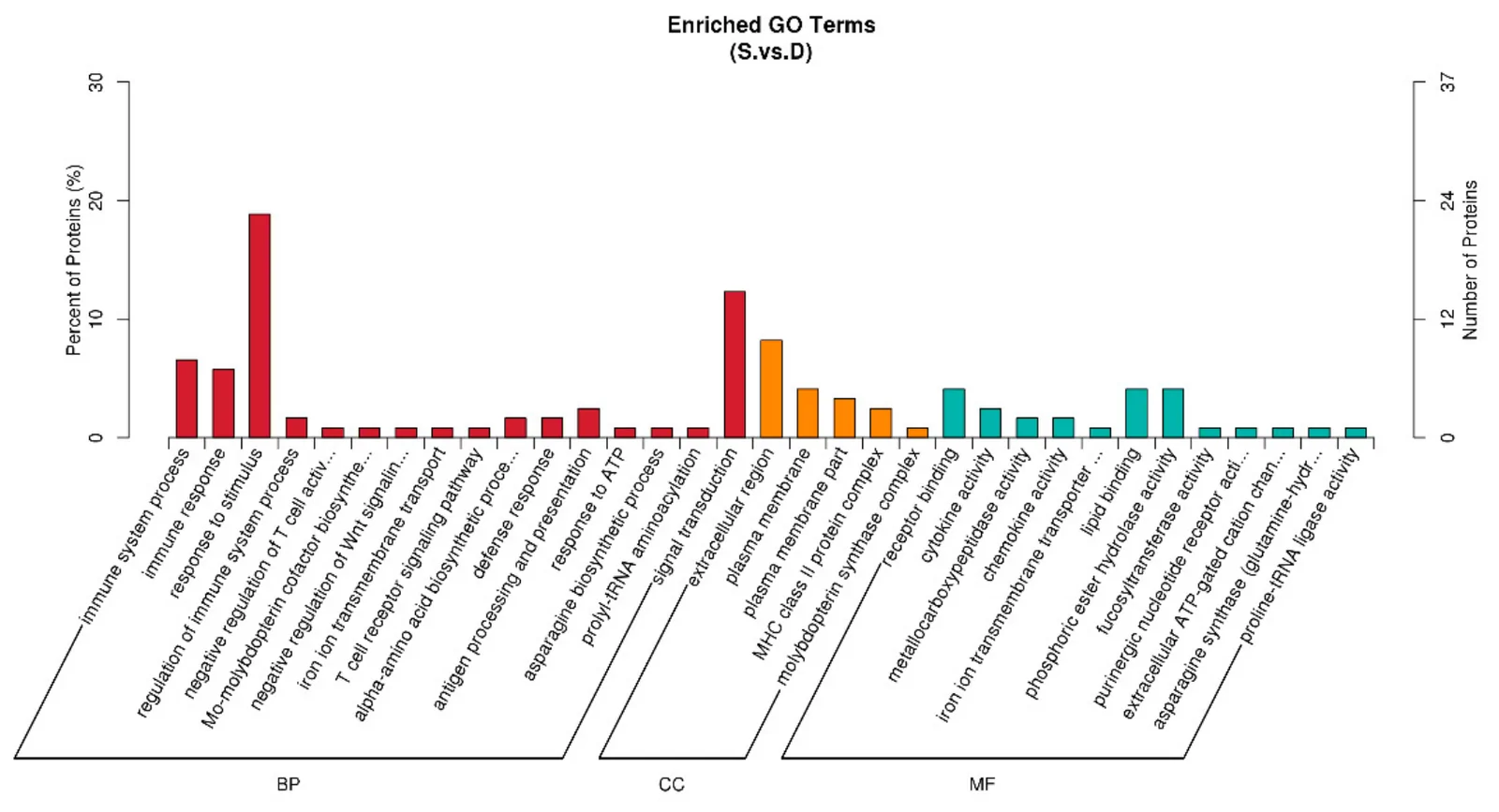
GO enrichment analysis of differentially expressed proteins between the supplementary-feeding group (S) and control group (D). The histogram illustrates the significantly enriched biological process (BP), cellular component (CC), and molecular function (MF) terms for differential proteins.

The significantly enriched GO terms (*p*-value < 0.05) are shown in Figure 10. Red, orange, and cyan represent the BP (biological process), CC (cell component), and MF (molecular function), respectively. The coordinate on the left is the percentage of the number of differentially expressed proteins annotated to a GO entry to the total number of differentially expressed proteins. The coordinate on the right is the number of differentially expressed proteins annotated.

The results showed that 16 proteins were involved in BP, 5 in CC, and 12 in MF. The specific functions of proteins involved in BP include immune regulation (37.70%), signal transduction (13.93%), and metabolic processes (5%). The proteins involved in the CC are extracellular (8.20%) and plasma membrane (7.38%). The proteins related to MF were mainly catalytic activity (14.75%) and a small part of lipid and receptor binding (8.20%).

**Figure 11:**
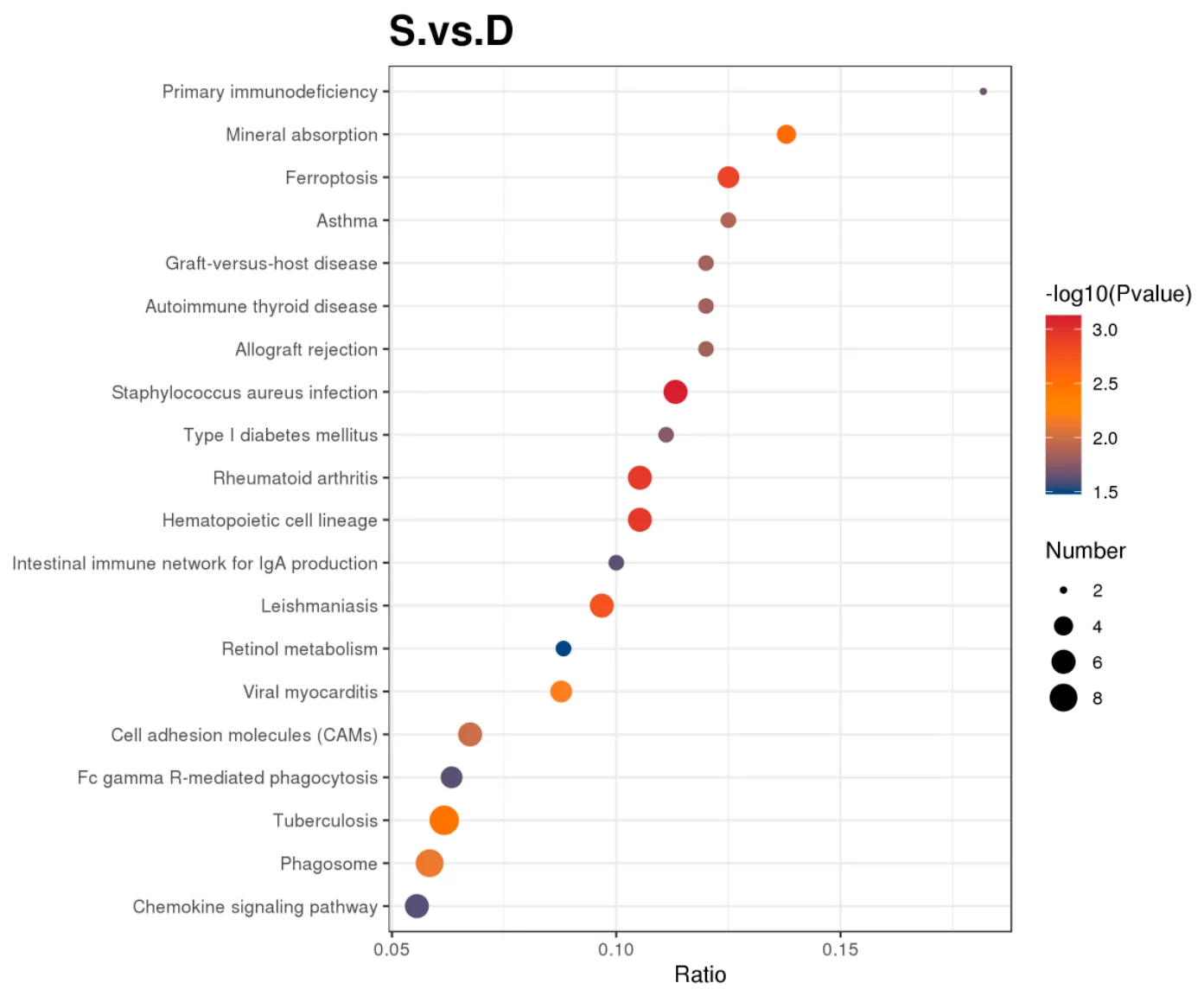
KEGG pathway enrichment bubble plot for differentially expressed proteins between the supplementary-feeding group (S) and control group (D). Each bubble represents an enriched KEGG pathway; bubble color indicates −log_10_(*p*-value), and bubble size denotes the number of enriched differential proteins.

The differentially enriched KEGG pathways included Staphylococcus aureus infection, hematopoietic cell lineage, rheumatoid arthritis, ferroptosis, leishmaniasis, mineral absorption, tuberculosis, viral myocarditis, phagosome, and cell adhesion molecules (CAMs). Figure 11 shows a KEGG enrichment bubble of the top 20 differentially expressed proteins.

**Figure 12:**
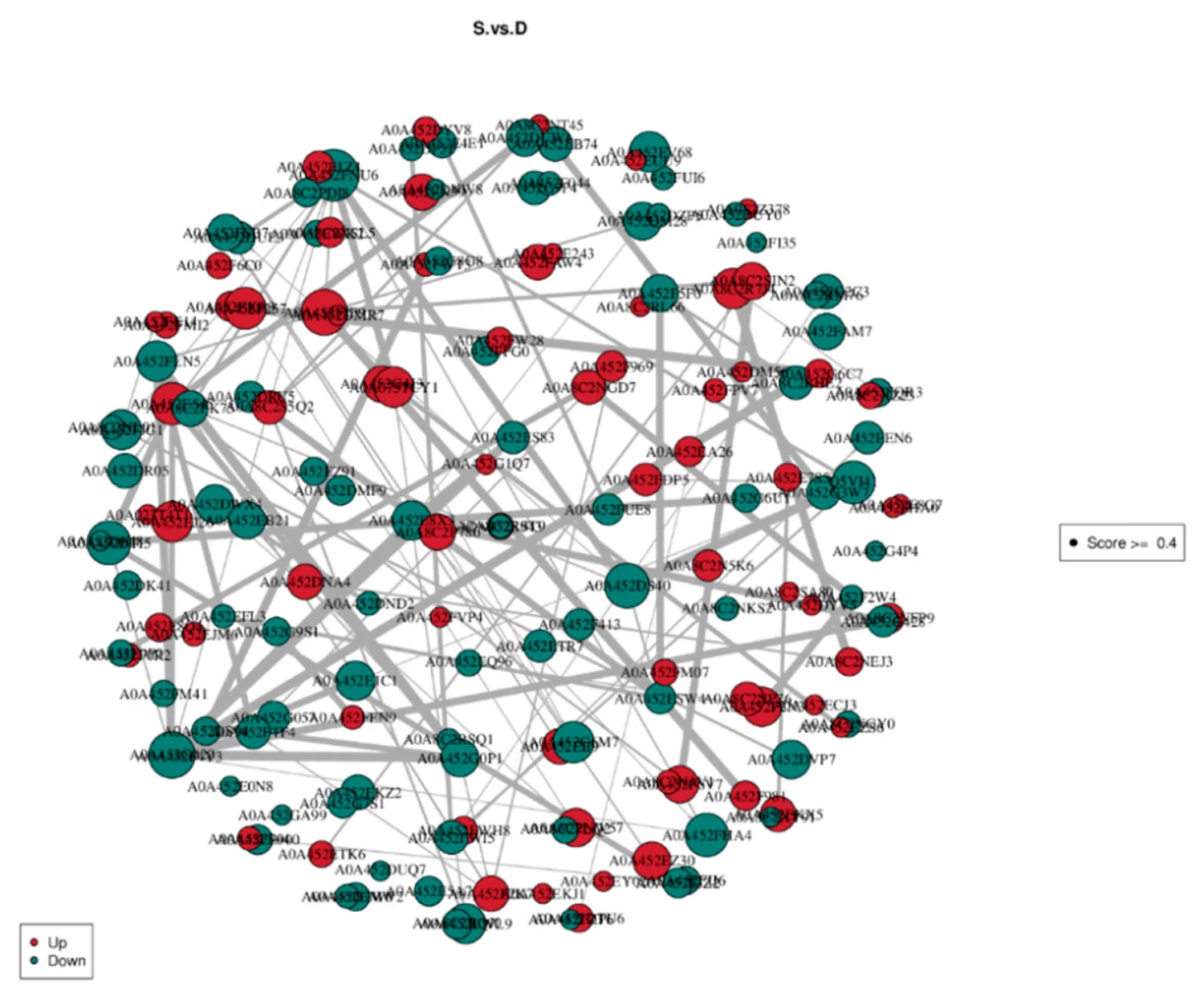
Diagram of the protein interaction network. Each node in the interaction network represents a protein: the larger the node, the more proteins it interacts with. The node color indicates the expression level of a single protein in the comparison pair. Red indicates a significantly high level of expression, and green indicates a significantly low level of expression.

The network diagram (Figure 12) shows the interactions between the |FC| TOP50 differential proteins. The proteins with a large number of interconnections and larger points (RBP4, KRTAP3-1, ITGAX, RAF1, AGT, DKK3, DUSP23, S100A3, KRTAP27-1, IL16, IGF2, KAP13-3, and ITGB2) were selected as key proteins.

### 3.5. Effects of Early Compound Nutritional Intervention on the Skin Metabolome of Lambs

**Figure 13:**
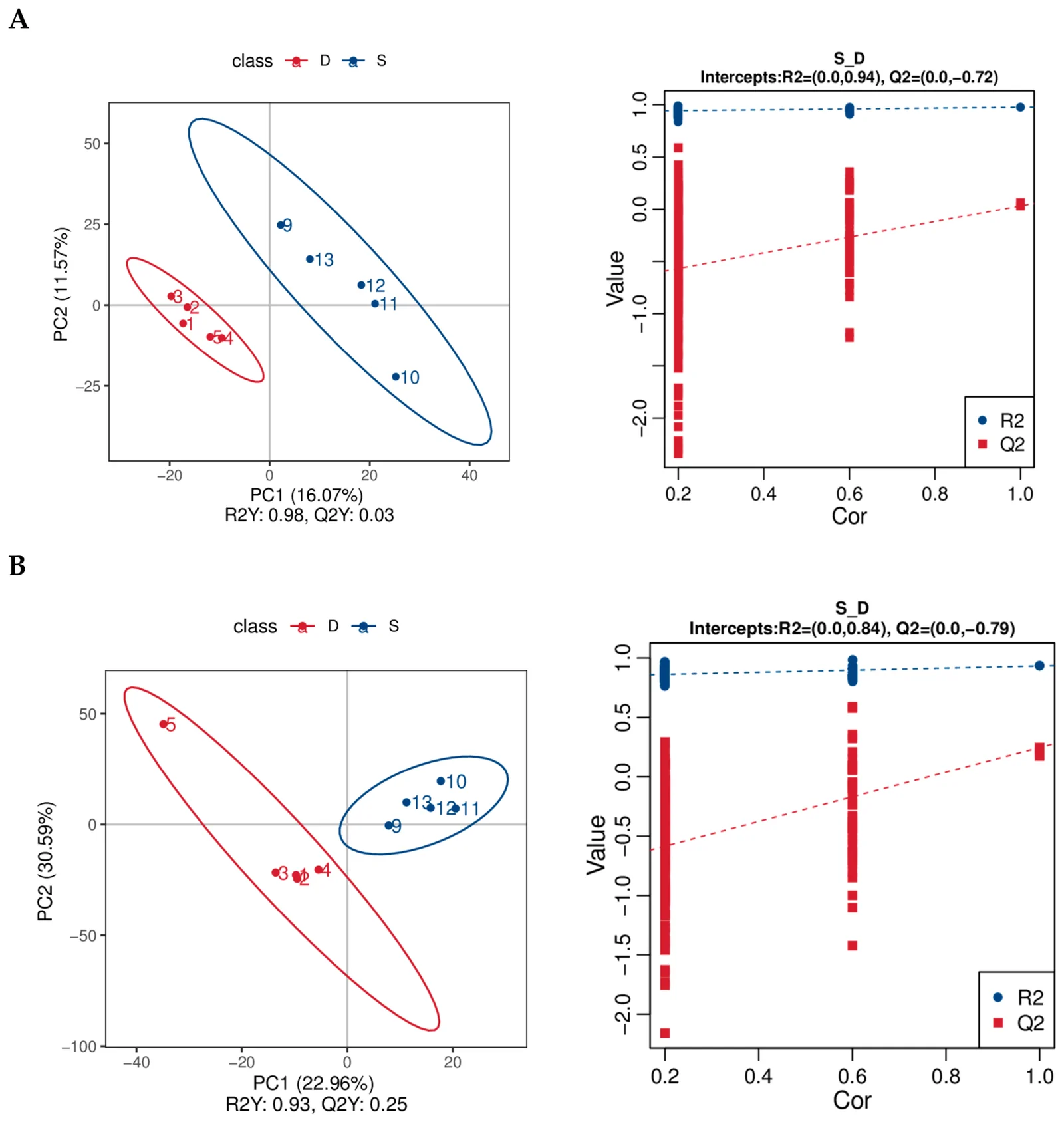
PLS-DA score of skin metabolites in two groups of lambs (in positive and negative ion modes). (**A**,**B**) represent the PLS-DA score and ranking verification diagrams of the skin metabolome in positive and negative ion modes, respectively.

As shown in Figure 13, (the positive and negative ion mode), the R2 of the model is greater than the Q2 value, and the intercept between Q2 and the Y axis is <0, indicating no overfitting phenomenon in the model. This model is suitable for describing the sample and for post-analysis to identify differential metabolites in the skin of the two lamb groups.

**Figure 14:**
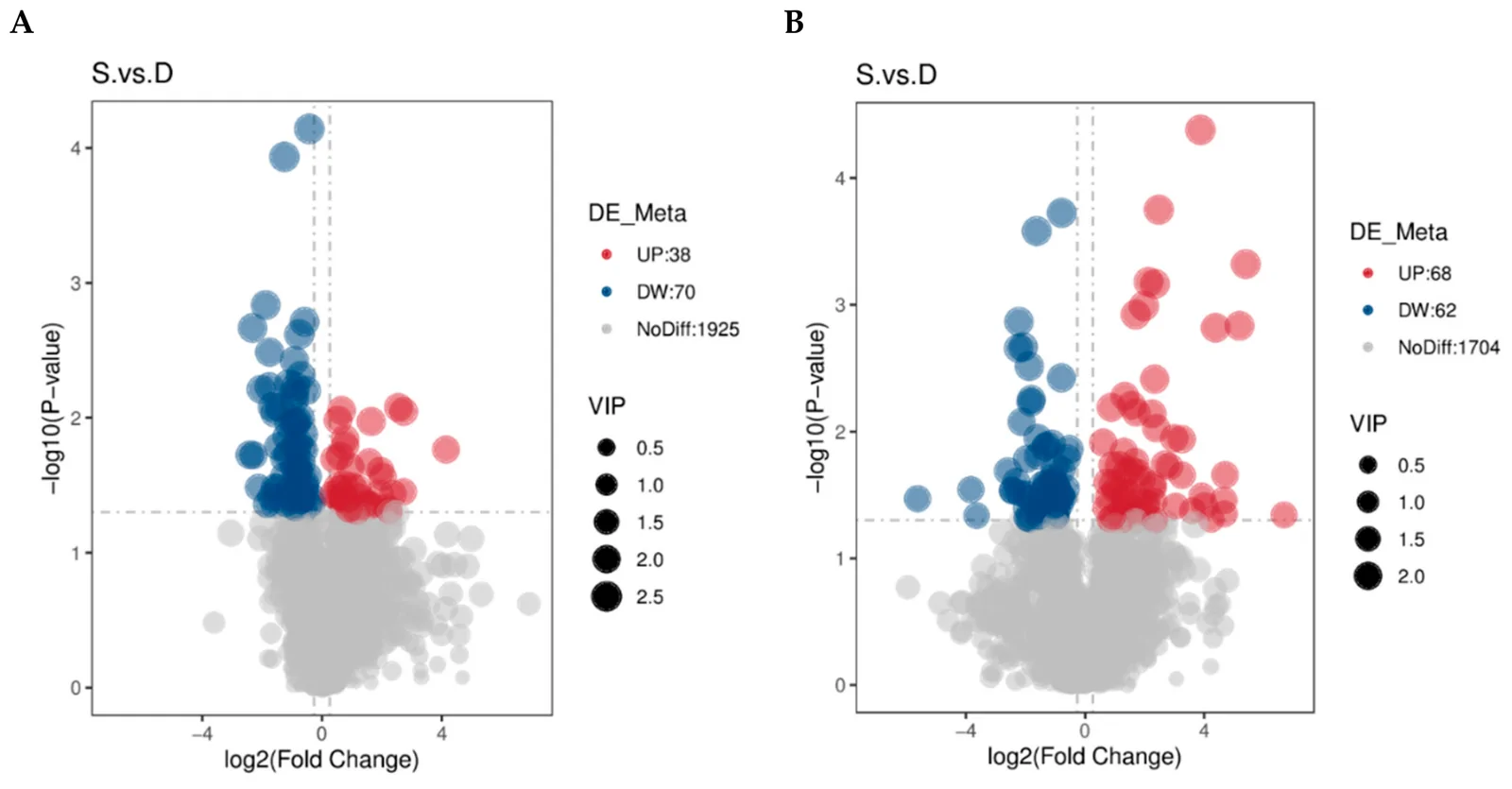
Volcano diagram of differential metabolites in the skin of two groups of lambs (in positive and negative ion mode). (**A**,**B**) represent the volcano plots of differential metabolites of the skin in positive and negative ion modes, respectively.

As shown in Figure 14 (VI 1.0, FC1.2, and *p*-value < 0.05), the 3867 metabolites contained 238 differential metabolites. The differential metabolites included 106 significantly up-regulated (such as L-lysose, arginine, cysteine, serine, methionine, proline, glycerophosphate (18:1 (9Z)/18:0), and glycine deoxycholic acid in the skin) and 132 significantly down-regulated (such as betaine, choline phosphate, aspartic acid, glutamine, monoacylglycerol (0:0/22:1(13Z)/0:0), and tetradecyl thioacetic acid).

**Figure 15:**
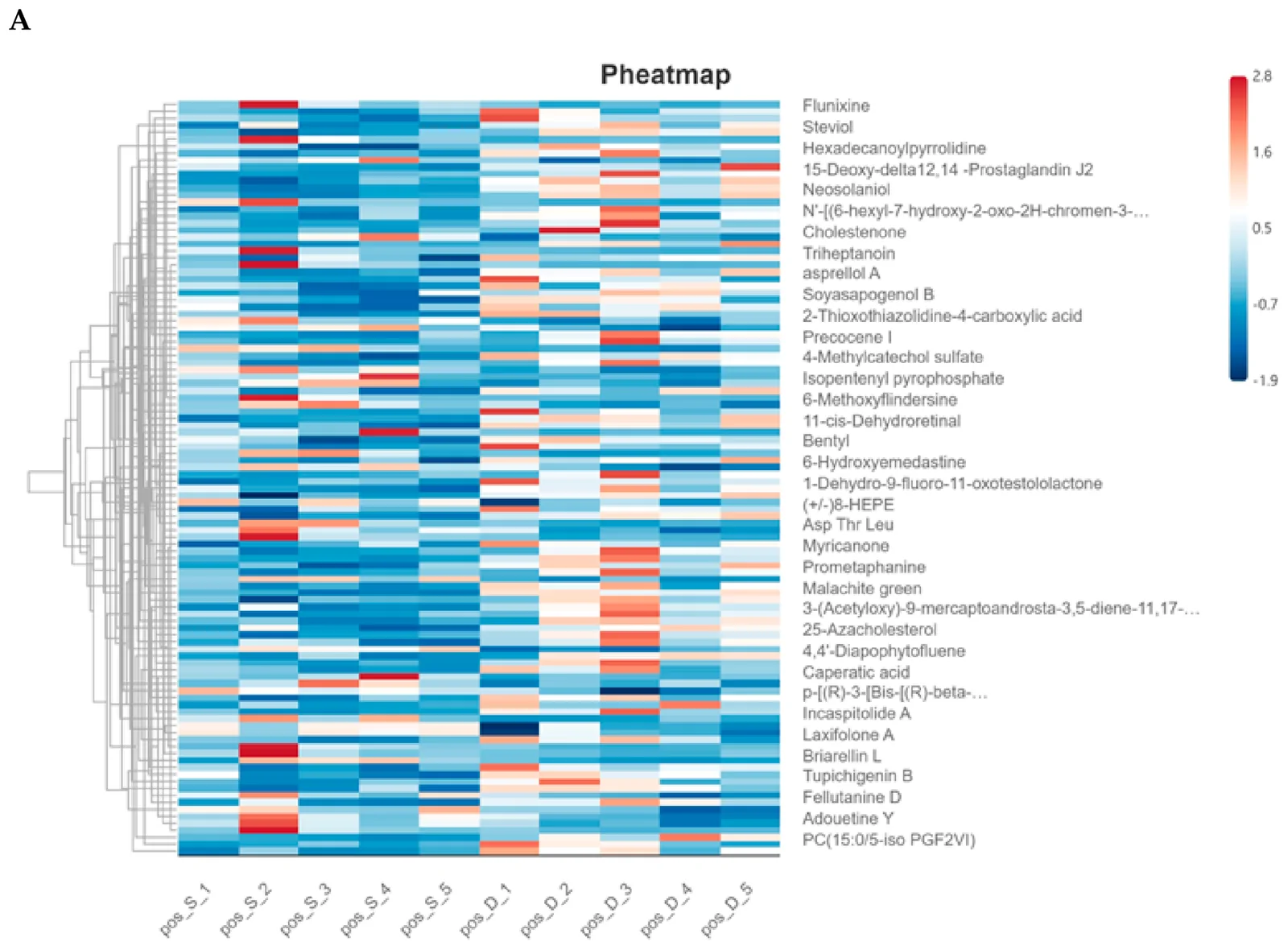

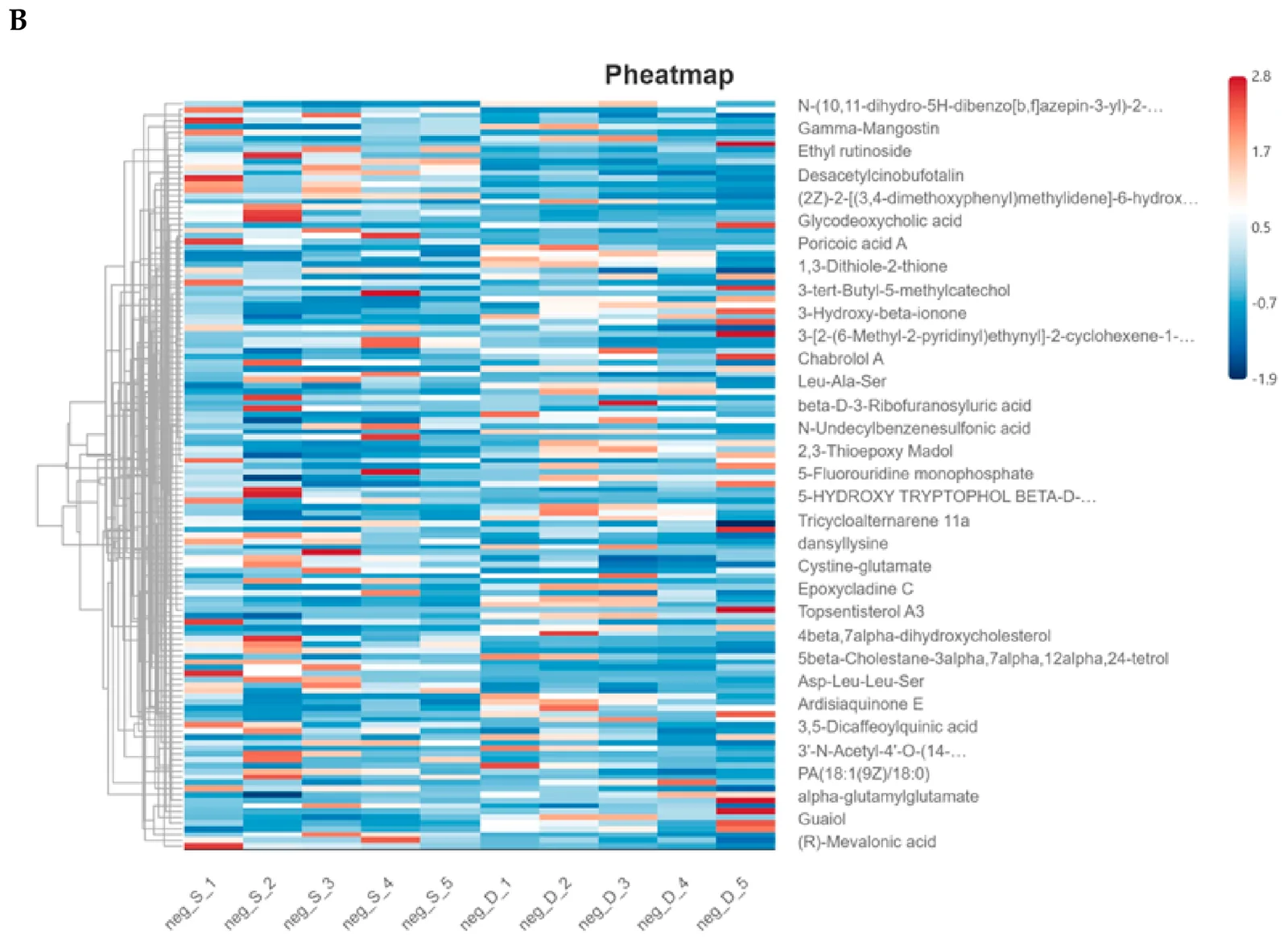
KEGG enrichment bubble diagram (positive and negative ion modes) of lamb skin metabolome in the two groups. (**A**,**B**) represent KEGG enrichment bubble plots of the skin metabolome in positive and negative ion modes, respectively.

The KEGG bubble diagram shows the top 20 pathways (Figure 15). The metabolic pathways were highly enriched and statistically significant in the positive ion mode. In contrast, the negative ion mode showed enrichment of the drug metabolism-cytochrome P450 pathway and the dopaminergic synapse pathway, each enriched by only one metabolite (with high enrichment levels and distinct).

## 4. Discussion

The growth and development of skin hair follicles in cashmere goats require a large amount of amino acids, energy, and trace elements. Thus, the addition of rumen-protecting amino acids and zinc to the diet of cashmere goats improved the growth of cashmere goats by promoting the development of secondary hair follicles [7,14]. Amino acids are the basis for the synthesis of related proteins and cashmere production in cashmere goats. Hence, the increase in protein intake in cashmere goats affected the synthesis and metabolism of cashmere. This study showed that adding appropriate amounts of pellet feed and rumen-protected methionine to the diet of cashmere goats significantly improved the density of secondary hair follicles. Therefore, adequate intake of protein and related amino acids could improve hair follicle development, consistent with the above-mentioned findings.

Cashmere production performance directly affects the economic benefits and sustainable development of cashmere goat breeding. Thus, cashmere production performance should focus on yield quality. The key indicator of cashmere quality is its fineness. The lower the fineness, the higher its value. However, limiting the nutritional intake of cashmere goats reduces cashmere yield because the body prioritizes maintaining basic nutritional needs for survival. In contrast, a high nutrient level results in thicker cashmere [15]. Increasing dietary nutrient levels improved cashmere yield and thickness [11]. In addition, the cashmere performance of younger goats was more susceptible to nutrient levels. In another study, a 10% dietary protein increased the cashmere of goats without thickening and promoted the growth of cashmere [16]. Therefore, it is necessary to find an optimal feeding amount to improve the yield and quality of cashmere. In addition, feeding cashmere goats with amino acids and other nutrients can promote the reduction in cashmere fineness [17]. The addition of rumen-protected cysteine, methionine, or both in the diet of goats could increase the length of cashmere and reduce its fineness, indicating that rumen-protected cysteine and methionine could promote cashmere growth [12]. However, compound nutritional intervention increased the fineness of cashmere slightly, with no significant difference between the two groups of lambs. This result may indicate that the supplemental rumen-protected methionine was below the optimum levels for increased yield and cashmere fineness.

Primary hair follicles germinate wool; secondary hair follicles germinate cashmere. Secondary follicle density and cashmere yield positively correlated [18]. Thus, the growth and development of secondary hair follicles can directly affect the quality of cashmere, and the density of secondary hair follicles determines the yield of cashmere. Therefore, the density of hair follicles and cashmere production are correlated. Previous studies have shown that the number of secondary hair follicles in cashmere goats is positively correlated with cashmere yield and negatively correlated with cashmere fineness [19]. Other studies have reported a negative correlation between the coarse hair fineness of rex rabbits, the fineness of beaver and sea otters, and the hair density [20,21], consistent with the results of this study. There is a significant negative correlation between secondary hair follicle density and cashmere fineness (*p* < 0.05), probably because, under the same nutritional level, increasing the density of secondary hair follicles reduces the material used by each hair follicle to synthesize cashmere. This hypothesis may explain how hair follicle density can affect cashmere fineness. Data on cashmere yield were beyond the scope of this study.

The transcriptome is the set of RNAs transcribed from a specific tissue or cell at a given time or state, primarily including mRNA and non-coding RNAs. Thus, studying all mRNAs in specific tissues is the basis for predicting gene function and interpreting their structure, a significant step toward understanding the development of animals at specific stages [22]. This experiment generated 42,759,213 and 42,753,040 reads from the control and the experimental groups, respectively. Gene expression levels are relatively uniform, with 14,408 co-expressed genes between the two groups. Then, 361 and 298 genes were expressed separately in the control and the experimental groups, respectively. Further analysis identified 1562 genes that were significantly different (681 up-regulated and 881 down-regulated). GO analysis showed that genes with large differences were mainly clustered in the cellular component. The top ten genes include keratin filament, intermediate filament, intermediate filament cytoskeleton, polymeric cytoskeletal fiber, supramolecular complex, supramolecular polymer, supramolecular fiber, cytoskeletal part, cytoskeleton, and plasma membrane part. The downstream function of these genes is for the synthesis of keratin fibers. Further, 191 differential genes were significantly enriched in 10 KEGG signaling pathways, including Staphylococcus aureus infection, cytokine–cytokine receptor interaction, chemokine signaling pathway, and protein digestion and absorption.

The Staphylococcus aureus infection pathway was enriched by KRT10, KRT18, KRT26, ITGB2, CSF1R, AKT1, COL6A6, COL6A2, COL6A1, and KRT10 genes related to keratin and hair follicle development. These genes are differentially expressed in animal epithelial cells, belong to the type 1 keratin family, and are important components of the cytoskeleton for signal transduction [23,24]. KRT10 is implicated in the differentiation and migration of hair follicle stem cells during hair follicle development and participates in epidermal keratinization in the skin [25,26]. In yaks, the KRT10 gene was associated with hair follicle development [27]. KRT18 is a member of the keratin family and can participate in the regulation of cell proliferation and differentiation [28]. Further, KRT10 and KRT18 are key proteins that affect the fineness of cashmere [29]. The KRT26 gene is associated with cashmere fineness [30]. ITGB2, a member of the integrin family, plays an important role in the proliferation, differentiation, and remodeling of stem cells [31].

In addition, the mRNA expression of ITGB2 is higher in coarse cashmere sheep than in finer ones [32]. In this study, KRT10, KRT18, and ITGB2 were significantly down-regulated in the Staphylococcus aureus infection pathway, while KRT26 was up-regulated. Based on the above reports and the hair follicle density results from this experiment, KRT26 may promote hair follicle growth and differentiation. In contrast, KRT10 and KRT18 inhibited hair follicle growth. Since there was no significant difference in cashmere fineness between the two groups of lambs in this experiment, the specific role of ITGB2 remains unknown.

In the cytokine–cytokine receptor interaction, CSF1R is related to hair follicle development. CSF1R is expressed in many goat breeds, is a key hub gene in the gene regulatory network, and is probably related to goat hair follicle development and the growth cycle [33]. AKT1 and other related genes show a strong correlation with the fineness of cashmere [34]. In the chemokine signaling pathway, AKT1 was significantly down-regulated. The PI3K-AKT signaling pathway is related to hair follicle regeneration [35], and AKT1 is among the components involved in the PI3K-AKT signaling pathway. These results collectively suggest that AKT1 may inhibit related chemokines and promote hair follicle differentiation, thereby affecting the fineness of cashmere.

In the protein digestion and absorption pathway, several collagen-related genes, including COL6A6, COL6A2, and COL6A1, were identified. COL1A1 is highly expressed in secondary hair follicle papilla cells [36], while COL6A1 and COL6A2 are candidate genes for hair follicle development [34]. Additionally, COL1A1 and COL1A2 may promote cashmere regeneration by regulating the differentiation of hair follicle stem cells [37]. The genes related to cashmere traits in cashmere goats have been identified, including the gene COL6A6 related to hair follicle development, and genes in the type IV collagen family, such as COL4A5, COL6A5, and COL6A6, in the EMC receptor interaction pathway [33]. The genes related to cashmere traits in cashmere goats were identified, including COL6A6, which is related to hair follicle development and genes in the type IV collagen family, such as OL4A5, COL6A5, and COL6A6, in the EMC receptor interaction pathway. These genes were considered candidate genes related to hair follicle growth and development. Studies have shown that extracellular matrix components are essential for maintaining the growth environment of hair follicle stem cells. Collagen is part of the matrix composition and may affect hair follicle growth by providing a microenvironment for hair follicle stem cells. In summary, the KRT10, KRT18, KRT26, ITGB2, CSF1R, AKT1, COL6A6, COL6A2, and COL6A1 genes identified in this experiment are candidates for hair follicle development and the regulation of cashmere fineness in cashmere goats.

Analysis of the skin proteome can identify key proteins involved in hair follicle development. Then the differential proteins and their interactions can be further explored to reveal the molecular mechanism of hair growth in hair follicles. Then the relevant functional proteins can be identified and combined with cashmere production performance for correlation analysis. This experiment identified 9589 proteins in the two lamb groups using DIA quantitative proteomics. The genes encoded 198 differentially expressed proteins (87 significantly up-regulated and 111 significantly down-regulated). After GO and KEGG enrichment analyses, 61 proteins were identified, which were enriched in 10 metabolic pathways (*p*-value < 0.05). These pathways include the Staphylococcus aureus infection pathway, hematopoietic cell lineage pathway, rheumatoid arthritis pathway, ferroptosis pathway, leishmaniasis pathway, mineral absorption pathway, tuberculosis pathway, viral myocarditis pathway, phagosome pathway, and cell adhesion molecules pathway (CAMs). Further, the protein network revealed RBP4, KAP13-3, KRTAP3-1, KRTAP27-1, and DKK3 as significantly up-regulated key proteins related to hair follicle development; ITGB2 and DUSP23 were significantly down-regulated.

RBP4 is a member of the lipid carrier protein family, mainly responsible for the transport of retinol (vitamin A1). Vitamin A1 is a derivative of vitamin A, which plays an important role in the growth and differentiation of hair follicles. In this experiment, the upregulation of RBP4 may indirectly regulate hair follicle growth and development, increasing hair follicle density. This result is consistent with the higher follicle density in the experimental group, which was significantly higher than in the control. Animal hair is a natural protein grown from hair follicles, and keratin is the main protein component of cashmere. Keratin supports cell structure, is an important marker of skin cell differentiation, and can participate in metabolic regulation and stimulate epithelial cell growth-related signaling pathways [38]. Keratin genes (KRTs) and keratin-related genes (KAPs) are closely related to the growth and development of hair follicles [39]. Keratin-related genes KRT71 and KRT75 are associated with hair follicle development [40].

Thus, this study profiled four key keratin proteins (KRT1, KRT17, KRT10, and KRT18), which may be related to the fineness of cashmere goats [29]. Improving the nutritional level (especially protein) causes the goat to produce more amino acids for keratin synthesis, thereby increasing the growth and density of secondary hair follicles, which in turn increases cashmere production.

Protein KK3 is a recognized inhibitor of the Wnt/β-catenin pathway [41], and it inhibits the signal transduction of this pathway by binding to LRP/6 [42]. The DKK3 and Wnt/β-catenin signaling pathways involving TCF3, CCND1, and other positive regulatory factors exert antagonistic effects [43]. DKK3 also promotes adipogenesis and stem cell differentiation by inhibiting the expression of Wnt/β-catenin pathway-related genes and mitochondrial autophagy. In this experiment, diet supplementation significantly up-regulated DKK3. Therefore, DKK3 may promote the production of fat required for hair follicle development by inhibiting the Wnt/β-catenin signaling pathway, thereby promoting hair follicle development and increasing its density.

Another protein, ITGB2 of the Hippo signaling pathway, is related to the Wnt signaling pathway and can synergistically regulate hair follicle development [44]. ITGB2 is also expressed in the anterior skin transcriptome and may participate in the proliferation, differentiation, and remodeling of stem cells. In this study, ITGB2 and its mRNA were down-regulated in the transcriptome and proteome. Considering that the cashmere finer goat had more ITGB2 mRNA, ITGB2 could increase the number of secondary hair follicles without changing the precursor of cashmere fineness, that is, promoting the proliferation and development of hair follicles. Most research on the dual-specific phosphatase (DUSP) focuses on the field of disease immunity, with few studies on animals. Dual-specificity phosphatase 6 (DUSP6) is a part of the MAPK signaling pathway. The MAPK pathway plays an important role in the development of certain diseases and tissue and organ regeneration [45]. The MAPK pathway is related to hair growth [46,47], and DUSP23 is a member of the bispecific phosphatase family. Thus, the DUSP23 protein probably inhibits hair growth.

The 3867 metabolites contained 238 differential ones (106 significantly up-regulated and 132 significantly down-regulated metabolites). A KEGG analysis showed that 27 differential metabolites enriched 22 metabolic pathways. However, 15-Deoxy-delta12,14-Prostaglandin J2 and Thromboxane B2 (TXB2) of the arachidonic acid metabolism pathway were significantly down-regulated.

In the arachidonic acid metabolism pathway, 15-Deoxy-delta12,14-Prostaglandin J2 and TXB2 share a common precursor, PGH2. There are four important downstream metabolic pathways of PGH2 in this pathway. The first pathway is catalyzed by thromboxane-A synthase (TBXAS1) to convert to thromboxane A2 (TXA2), then to TXB2, and finally to 101-Dehydro-thromboxane B2. The second pathway involves the conversion of prostaglandin-H2 by D-isomerase to prostaglandin D2 (PGD2) and finally to 15-Deoxy-delta12,14-Prostaglandin J2. The third metabolic pathway is catalyzed by microsomal prostaglandin E synthase 1 (PTGES) into prostaglandin E2 (PGE2), which is then transformed into PGA2, PGC2, and PGB2. The fourth metabolic pathway is converted to prostaglandin F2α (PGF2α) catalyzed by rosamine/prostaglandin f2α synthase (PRXL2B) and 9,11-endoperoxide prostaglandin H2 reductase (PGFS).

Studies have found that prostaglandins can participate in the metabolism of human hair cells. The site of PGE2 and PGF2α transformation is the dermal papilla epithelial cells, which may be involved in the differentiation of hair follicles [48]. In hair follicle development, PGD2 can inhibit hair growth, while PGE2 and PGF2α can promote hair growth [49]. Furthermore, prostaglandin derivatives can promote the transformation of hair follicles from telogen to anagen, thereby improving related traits [50], consistent with the results of this study. In this study, deoxy-delta12,14-prostaglandin J2 and TXB2 were significantly down-regulated, implying that the PGH2 metabolic pathway leading to their synthesis is relatively inhibited. Although PGE2 and PGF2α did not reach significant up-regulation in metabolomic detection, their precursor substances showed an upward trend in the experimental group relative to the control group. Combined with the known biological roles reported in the previous literature, we infer that the reduced flux toward inhibitory metabolites may redistribute PGH2 substrate toward the synthesis of PGE2 and PGF2α. Thus, the arachidonic acid metabolism pathway may indirectly regulate the growth and development of hair follicles by regulating prostaglandin derivatives, which in turn affect hair follicle density and related cashmere production performance.

Integrating phenotypic, transcriptomic, proteomic and metabolomic datasets, dietary supplementation with rumen-protected methionine alleviates the deficiency of sulfur-containing amino acids inherent to high-altitude native pasture, providing sufficient precursor substrates for cutaneous keratin biosynthesis. Metabolically, adequate amino acid supply remodels the arachidonic acid metabolic microenvironment of skin tissue. The inhibitory lipid mediators 15-Deoxy-delta12,14-Prostaglandin J2 and TXB2 are significantly downregulated, thereby lifting the repression of hair follicle stem cell proliferation. This metabolic shift redirects the flux of PGH2 toward biosynthetic pathways, generating pro-folliculogenic PGE2 and PGF2α. The optimized cutaneous metabolic microenvironment further activates core developmental signaling pathways at the molecular level. Multi-omics conjoint analysis revealed coordinated alterations in homologous functional molecules. Transcriptomic data showed reduced expression of KRT10 and KRT18, which hinder hair follicle differentiation, while the follicle-promoting gene KRT26 was upregulated. Proteomic profiling identified abundant keratin-associated proteins (KAP13-3, KRTAP3-1, KRTAP27-1), whose synthesis relies heavily on sulfur-containing amino acids. Altered expression of AKT1 and DKK3 maintains the homeostatic balance of the PI3K-AKT and Wnt signaling cascades. Collagen genes COL6A1, COL6A2 and COL6A6 remodel the extracellular matrix to foster a favorable niche for hair follicle stem cells. Meanwhile, the concurrent downregulation of ITGB2 at both transcriptional and translational levels partly accounts for the slight decrease in cashmere fineness observed in the supplemented group.

These multi-tiered molecular shifts eventually induced divergent hair follicle and cashmere traits between treatment groups. Primary hair follicles complete full differentiation prior to birth and barely respond to short-term nutritional adjustments. In contrast, secondary follicles stay within an active proliferative phase post-lambing and rely heavily on exogenous nutrients, which explains why supplemental feeding only raised mature secondary follicle density with no detectable change to primary follicles. Greater secondary follicle density further boosted the lambs’ cashmere-producing capacity. Unlike earlier low-altitude research centered on maternal supplementation during gestation, the present work unravels a specific nutritional regulatory pathway in newborn lambs subjected to high-altitude nutrient limitation. It establishes a full mechanistic chain connecting dietary nutrient provision to cashmere-associated follicle phenotypes through metabolic reprogramming and multi-layered molecular modulation.

Notably, the supplementary-feeding regimen in this trial incorporated multiple nutritional additives, including pellet feed, milk powder, rumen-protected methionine, and lactobacillin tablets, whereas lambs in the control group received only maternal milk. Given this experimental setup, we cannot tease apart the individual contributions of energy, protein, sulfur-containing amino acids and probiotics to secondary-hair-follicle development and cashmere-related traits in Northwest Xizang white cashmere goats. Furthermore, only one dosage of rumen-protected methionine was applied in the present study without gradient-dose groups, so definitive conclusions cannot be drawn about whether this dosage is optimal for improving cashmere fineness. Future single-factor feeding trials with gradient methionine treatments are therefore required to screen the key nutrient and determine the optimal supplemental methionine level for lambs of this breed under high-altitude nutrient-deficient stress.

## 5. Conclusions

This experiment showed that early compound nutritional intervention of white cashmere goat lambs in northwest Xizang can promote the proliferation and development of secondary hair follicles without affecting the fineness of cashmere and improve the relevant hair follicle density indicators.

The transcription, protein, and metabolomics data of lamb skin tissue showed that KRT10, KRT18, KRT26, ITGB2, CSF1R, AKT1, COL6A6, COL6A2, and COL6A1 are the candidate genes related to hair follicle development and cashmere fineness. The key proteins related to hair follicle development included RBP4, KAP13-3, KRTAP3-1, KRTAP27-1, DKK3, ITGB2, and DUSP23. The differential metabolites, 15-Deoxy-delta12,14-Prostaglandin J2 and TXB2, may indirectly regulate the growth and development of hair follicles by regulating prostaglandin derivatives through the arachidonic acid metabolism pathway. This reaction may, in turn, affect hair follicle density and related cashmere production performance.

This study constructs a mechanistic link connecting nutritional supplementation, skin metabolic reprogramming and hair-follicle phenotypes in young cashmere goats under high-altitude conditions. It provides theoretical support for optimizing feeding strategies to improve cashmere-producing performance in alpine pastoral regions.

## Figures and Tables

**Table 1:** Feed and additive amounts provided at different experimental stages (g/lamb per feeding).

Stage	Dosage of Feed and Additives			
	Pellet Feed	Methionine	Lactobacillin Tablets	Milk Powder
Test 1–20 d	200	3.75	0.9	50
Test 20–30 d	300	3.75	0.9	30
Test 30–60 d	450	3.75	0.9	-

**Table 2:** Ingredients and nutrient levels of the basal diet (%).

Ingredients	Content (%)	Nutrient Levels	Content (%)
Corn	59.30	Metabolic energy (MJ/kg)	12.82
Soybean meal	8.10	Dry matter	87.92
Expanded soybean	10.0	Crude protein	18.02
Cotton meal	5.00	Crude fat	5.10
DDGS	13.20	Crude ash	7.04
Calcium carbonate	2.00	Neutral detergent fiber	12.85
Calcium hydrogen phosphate	0.80	Acid detergent fiber	5.89
Salt	0.60	Calcium	1.27
Premix	1.00	Phosphorus	0.67

Note: The premix provides 15,000 IU of VA, 3000 IU of VD3, 10 IU of VE, 10 mg of copper, 25 mg of iron, 20 mg of zinc, 20 mg of manganese, 0.5 mg of iodine, and 0.4 mg of selenium per kilogram of granular material. 2. ME was a calculated value, while the others were measured values.

**Table 3:** Effect of early compound nutritional intervention on the hair follicle density of lambs.

Items	Control Group	Test Group	*p*-Value
Primary hair follicle density (number/mm^2^)	5.44 ± 0.71	5.79 ± 0.27	0.664
Secondary hair follicle density (number/mm^2^)	23.20 ± 4.91 ^b^	48.22 ± 6.05 ^a^	0.012
Total hair follicle density (number/mm^2^)	28.64 ± 5.16 ^b^	54.01 ± 6.22 ^a^	0.014
S/P value	4.24 ± 0.77 ^b^	8.22 ± 1.00 ^a^	0.014

Note: ^a,b^ Means with different lowercase superscripts are significantly different (*p* < 0.05).

**Table 4:** Effects of early compound nutritional intervention on the cashmere production of lambs.

Items	Control Group	Test Group	*p*-Value
Cashmere fineness (μm)	13.63 ± 0.23	13.68 ± 0.28	0.765
Wool length (cm)	8.64 ± 0.23 ^B^	10.15 ± 0.28 ^A^	0.006

Note: ^A,B^ Means with different lowercase superscripts are significantly different (*p* < 0.01).

**Table 5:** Summary table of the skin transcriptome sequencing data.

Samples	Total Clean Reads	Number of Total Mapped Reads (Rate)	Number of Unique Mapped Reads (Rate)	Q30/%	GC Content/%
D1	43,049,158	41,890,944 (97.31%)	39,907,652 (92.7%)	96.55	49.6
D2	42,700,946	41,650,543 (97.54%)	39,517,906 (92.55%)	96.52	49.67
D3	41,006,216	39,908,528 (97.32%)	38,030,020 (92.74%)	96.64	49.69
D4	41,821,650	40,867,071 (97.72%)	38,930,650 (93.09%)	96.79	49.09
D5	42,419,866	41,389,738 (97.57%)	39,225,303 (92.47%)	96.73	49.27
S1	40,901,840	39,897,912 (97.55%)	38,072,009 (93.08%)	96.69	48.93
S2	39,939,216	38,679,461 (96.85%)	36,762,269 (92.05%)	96.73	49.29
S3	42,562,638	41,557,717 (97.64%)	39,209,368 (92.12%)	96.79	49.67
S4	42,593,146	41,553,288 (97.56%)	39,306,687 (92.28%)	96.66	49.68
S5	44,892,676	43,631,432 (97.19%)	41,383,108 (92.18%)	96.58	49.02

Note: Q30: percentage of bases with a Qphred value greater than 30 in the total base. D1–D5 represent individuals from the control group; S1–S5 represent individuals from the compound nutritional intervention group.

**Table 6:** Results of KEGG enrichment analysis of skin transcriptome.

Pathway Name	Padj	Gene Name
Staphylococcus aureus infection	0.00	*KRT10*, *LOC102176997*, *LOC108634681*, *LOC102176457*, *KRT18*, *MASP1*, *KRT26*, *ITGB2*, *LOC100860813*, *LOC102180664*, *LOC102180595*, *LOC102176726*, *C3AR1*, *LOC102190074*, *SELPLG*, *LOC102176161*, *CFB*, *LOC102189356*, *LOC102179881*, *LOC102187356*
Hematopoietic cell lineage	0.00	*CSF1*, *CD5*, *LOC102179433*, *CD44*, *GP5*, *LOC102181422*, *FCER2*, *IL1R2*, *CSF1R*, *IL5RA*, *LOC100860813*, *CD36*, *LOC102180664*, *LOC102174471*, *LOC102175833*, *CD24*, *IL4R*, *LOC102182494*, *CD37*, *LOC102189356*, *LOC102187356*
Viral protein interaction with cytokine and cytokine receptor	0.00	*CXCR2*, *CSF1*, *CXCR1*, *ACKR3*, *LOC102180231*, *LOC102171053*, *LOC102170310*, *IL22RA1*, *CCL26*, *CCL5*, *LOC102175889*, *CSF1R*, *IL37*, *CXCL8*, *LOC102169211*, *CCL22*, *LOC102175959*, *LOC102182395*, *CCL28*, *CXCL14*
Cytokine–cytokine receptor interaction	0.01	*CXCR2*, *CSF1*, *CD40*, *IL33*, *TNFRSF11B*, *CXCR1*, *ACKR3*, *LOC102180231*, *LOC102171053*, *LOC102170310*, *IL22RA1*, *INHBB*, *CCL26*, *CCL5*, *LOC102175889*, *IL1R2*, *CSF1R*, *IL37*, *IL5RA*, *CXCL8*, *LOC102169211*, *IL15RA*, *CCL22*, *IL4R*, *CD27*, *OSMR*, *IL17C*, *TNFSF13*, *LOC102175959*, *EDA2R*, *BMP8A*, *LOC102182395*, *IL25*, *GHR*, *CCL28*, *IFNGR2*, *CNTFR*, *CXCL14*, *LOC102174566*, *NGFR*
Chemokine signaling pathway	0.01	*CXCR2*, *CXCR1*, *NCF1*, *LOC102171053*, *PIK3CD*, *LOC102170310*, *WAS*, *CCL26*, *LOC102176514*, *FGR*, *CCL5*, *LOC102175889*, *CXCL8*, *LOC102169211*, *ARRB1*, *GNG13*, *CCL22*, *HCK*, *PREX1*, *PLCB2*, *SHC1*, *LOC102182395*, *DOCK2*, *LOC108638023*, *CCL28*, *AKT1*, *CXCL14*, *PIK3R5*
Rheumatoid arthritis	0.01	*CSF1*, *LOC102171053*, *CCL5*, *LOC102175889*, *ITGB2*, *LOC100860813*, *LOC102180664*, *CXCL8*, *TNFSF13*, *ATP6V0A4*, *LOC102182395*, *ATP6V1E1*, *TLR2*, *ATP6V1B1*, *CTSV*, *LOC102189356*, *LOC102187356*
Malaria	0.01	*CD40*, *THBS4*, *MET*, *ACKR1*, *LOC102175889*, *ITGB2*, *CD36*, *CXCL8*, *THBS1*, *SELE*, *TLR2*, *LOC102183506*
Complement and coagulation cascades	0.02	*F3*, *PLAT*, *LOC108634681*, *ITGAX*, *MASP1*, *ITGB2*, *LOC102175833*, *F2R*, *F13A1*, *LOC102183974*, *PLAUR*, *C3AR1*, *CFB*, *SERPINF2*, *PROCR*
Protein digestion and absorption	0.04	*COL6A6*, *COL6A2*, *COL6A1*, *COL7A1*, *COL13A1*, *COL17A1*, *COL6A3*, *COL24A1*, *SLC9A3*, *COL5A3*, *COL3A1*, *COL27A1*, *SLC1A5*, *ATP1B4*, *COL5A1*, *COL5A2*, *ATP1B2*, *LOC102183671*

## Data Availability

The original contributions presented in this study are included in the article. Further inquiries can be directed to the corresponding authors.

## Appendix A

**Table A1:** Primer sequences used for quantitative real-time PCR.

Target/Reference Gene	Primer	Primer Sequence (5′ → 3′)
*GAPDH* (reference gene)	F-primer	CCGTAACTTCTGTGCTGTGCC
	R-primer	TGAAGGGGTCATTGATGGCAAC
LOC102185525	F-primer	GCTCACACCTTCAGGTTGCT
	R-primer	TTGCCAAAACTGCAAGCGTTC
LOC102178529	F-primer	AATCGACCCACAGGAGGAAC
	R-primer	ATAAAGACCACAGCACCTCCA
*PPP1R17*	F-primer	AGACTGGACAAGCTAGACCCT
	R-primer	TGTTCTGAAAGGACGTCTGGTA
*TRIM6*	F-primer	GCTTCGAGTGTTTAGAGAGCTG
	R-primer	CTTCCAGATGAGACCCGGAC
*TCN1*	F-primer	AGGTGTGCATCGGGTAAAT
	R-primer	AGGACGTTGGCATCTTGGAA
*NTSR1*	F-primer	TCTTACGTGCCGTGGTCATC
	R-primer	GAAGTCGTAGAGGAACGGGG
LOC102180308	F-primer	TCTTACGTGCCGTGGTCATC
	R-primer	GAAGTCGTAGAGGAACGGGG
